# Stepwise Evolution Improves Identification of Diverse Peptides Binding to a Protein Target

**DOI:** 10.1038/s41598-017-12440-1

**Published:** 2017-09-21

**Authors:** Victor I. Lyamichev, Lauren E. Goodrich, Eric H. Sullivan, Ryan M. Bannen, Joerg Benz, Thomas J. Albert, Jigar J. Patel

**Affiliations:** 1 Roche Madison, 500 S Rosa Rd, Madison, WI 53719 USA; 2 Roche Pharmaceutical Research and Early Development, Therapeutic Modalities, Roche Innovation Center Basel, Grenzacherstrasse, 4070 Basel, Switzerland

## Abstract

Considerable efforts have been made to develop technologies for selection of peptidic molecules that act as substrates or binders to a protein of interest. Here we demonstrate the combination of rational peptide array library design, parallel screening and stepwise evolution, to discover novel peptide hotspots. These hotspots can be systematically evolved to create high-affinity, high-specificity binding peptides to a protein target in a reproducible and digitally controlled process. The method can be applied to synthesize both linear and cyclic peptides, as well as peptides composed of natural and non-natural amino acid analogs, thereby enabling screens in a much diverse chemical space. We apply this method to stepwise evolve peptide binders to streptavidin, a protein studied for over two decades and report novel peptides that mimic key interactions of biotin to streptavidin.

## Introduction

The majority of therapeutics on the market fall into two general categories: (1) small molecules with a molecular weight below 500 Da, and (2) biologics, such as antibodies, with a molecular weight over 150 kDa. However, there remains a plethora of candidate therapeutic targets with extended binding sites that are considered “undruggable” by both small molecule therapeutics and complex biologics. Peptide-based molecules are an emerging class of drug compounds that can potentially bridge the gap between small and complex molecules. Peptides offer the structural diversity required for selective and high affinity interactions while maintaining lower production costs than protein-based pharmaceuticals^1^.

A significant number of peptidic compounds with favorable therapeutic properties are currently on the market. For example, the cyclic peptide cyclosporine A is an 11-residue peptide with drug-like cell permeability, oral bioavailability, and stability *in vivo*. Other clinically useful peptide-based drugs include echnicandins^2^, daptomycin^3^, and actinomycin D^4^. Interestingly, these and other naturally occurring peptides often possess non-natural modifications including varied backbone stereochemistry, N-methylation, and macrocyclic structures which contribute to their potent activity and favorable pharmacokinetic properties^5^.

The need to discover peptidic molecules with optimal therapeutic properties has spurred the development of several platforms that aim to imitate natural selection. These platforms rely on building diverse libraries and either a screening or selection technique to identify a phenotype of interest^6^. Selection-based platforms, such as phage, ribosome, or mRNA display generally couple genotype with phenotype to link function with DNA survival such that DNA coding for only the fittest variants will be recovered. Display-based libraries typically identify molecules with high target-binding affinities through multiple rounds of selection.

Despite improvements to classical display techniques such as the Flexizyme^7,8^ and RaPID^9^ technologies, the need still exists for (1) more diverse libraries containing modifications (e.g., N-alkylation, D-stereochemistry, and cyclization) that are commonly found in natural bioactive peptide molecules, (2) methods that select for molecules with inherent properties such as proteolytic stability and cell permeability, and (3) the ability to rapidly iterate and rationally mature a “hit” to a “lead” by exploring a pre-defined chemical space to further optimize therapeutic characteristics.

Here we report a digital light-directed array technology^10–12^ to synthesize arrays containing peptides on an amine-functionalized slide. This technology uniquely combines four characteristics that could facilitate its use as a novel binder-discovery platform: (1) high feature density, resulting in 2.9 million unique molecules per array; (2) ability to accommodate broad chemical diversity, e.g., non-natural amino acids; (3) digitally controlled synthesis, allowing rapid iteration of library design; and (4) reproducible and highly sensitive screening. As a proof of concept, we used this platform to identify both known and novel L- and D-amino acid peptide binders to the well-characterized model target, streptavidin. Starting with a combinatorial 5-mer library, we identified various hotspot sequences that were evolved into larger peptides by a step-wise approach using rationally designed libraries. Further, we screened cyclic peptides with L- and D- amino acids to discover a 5-mer cyclic peptide that binds to streptavidin. The obtained results were confirmed by SPR analysis and co-crystallization with streptavidin.

## Results

### Stepwise evolution approach

**Figure 1:**
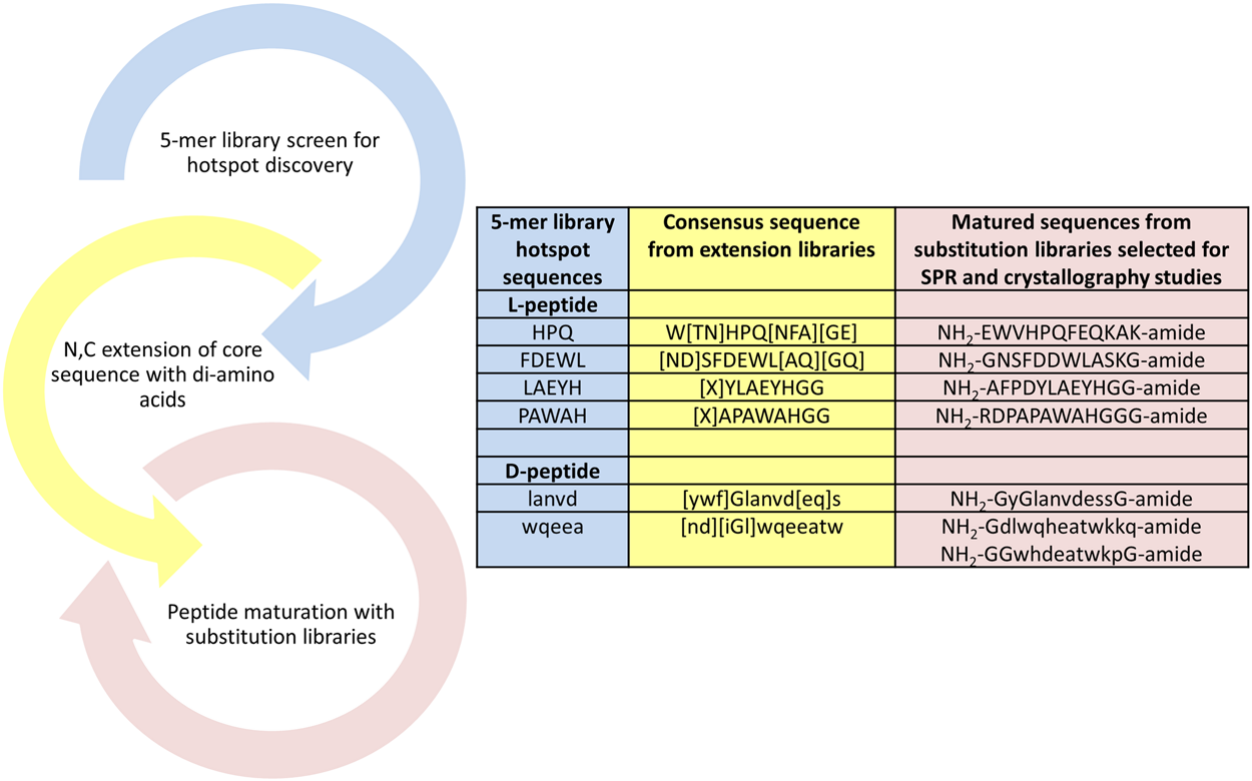
Schematic representation of the combined stepwise evolution and rational peptide array design approach. The inset table shows L- and D-peptide sequences (shown in uppercase and lowercase, respectively) selected at different steps of streptavidin binder evolution by using 5-mer, extension, and substitution libraries. Residues shown in [square brackets] are alternative amino acids for that position.

Figure 1 schematically shows the sequence of our stepwise approach to peptide binder discovery: (1) identification of “hotspot” sequences using comprehensive 5-mer library; (2) motif extension using libraries with an invariant sequence and all possible combinations of di-amino acids at its N- and C-termini; and (3) binder maturation using iterative libraries of all possible single- and double substitutions and deletions of peptide candidates selected in the first two steps.

### L-amino acid peptides binding to streptavidin

To identify hotspot sequences that bind to streptavidin (SA), we bound Cy5-SA to an array library of 2,476,099 5-mer L-peptides synthesized with 19 of 20 natural amino acids (excluding cysteine). The fluorescence signal intensity was aggregated across three independently synthesized arrays; then the 2,047 peptides with a signal-to-background ratio (S/B) >4 were selected. The data was further filtered to select 1,100 peptides for which signal intensities were highly correlated on all 3 arrays (the percentage of mean deviation to mean was <10%, see Supplementary dataset Table 2A). Most selected sequences (1,019 of 1,100) contained HP, PQ, or PM sequences: their presence would be expected in the well-known HPQ and HPM streptavidin binders^13–18^.

The remaining 81 non-HPQ sequences shown in Supplementary dataset Table 2B were analyzed using PEPLIB^19^. Several peptide sequence clusters were identified (Supplementary Fig. 1). The peptides FDEWL, LAEYH, and PAWAH were selected as representative sequences from distinct clusters, along with the abundant HPQ motif, as hotspot sequences for the next step of evolution (table inset Fig. 1). Each of these 4 sequences was extended from both the N- and the C-termini with all possible 160,000 combinations of L-amino acid dimers, using all 20 natural L-amino acids (see Materials and Methods). Streptavidin binding to these 4 new libraries exhibited an amino acid preference at both the N- and C-termini, as shown by the Logo plots (weblogo.berkely.edu) in Supplementary Figure 2. The table inset in Fig. 1 shows the consensus sequences identified with the extension libraries.

**Figure 2:**
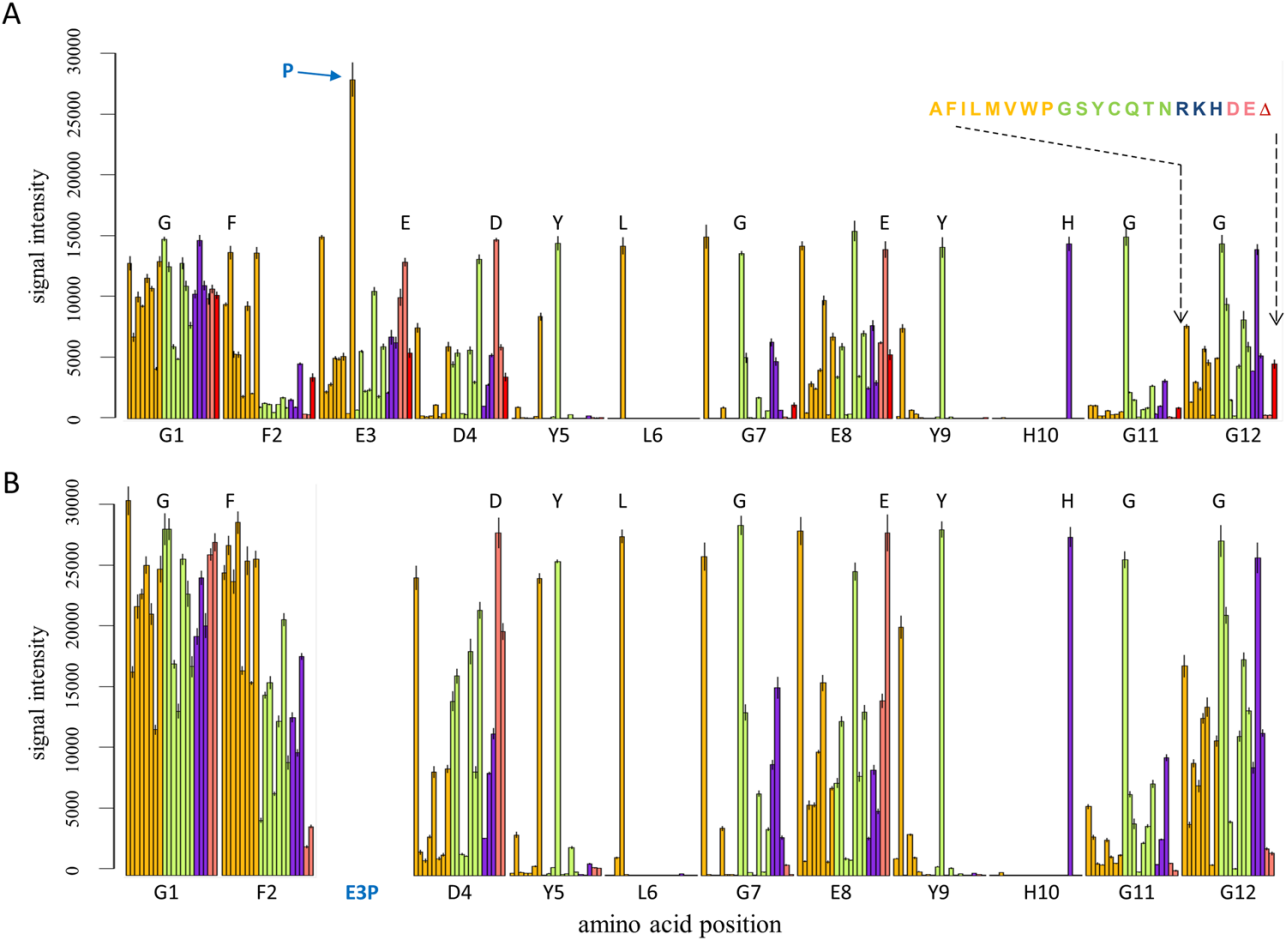
Single substitution plot for the GFEDYLGEYHGG peptide (**A**) and a double-substitution plot for its E3P variant, GFPDYLGEYHGG (**B**). Each peptide position is represented by 20 or 21 colored bars [one bar for each of the 20 amino acids, and a deletion (only in Panel 2 A)]; the height of each bar indicates the signal intensity. Bars corresponding to the “wild type” amino acid for each position are indicated by the single-letter amino acid code. The fixed E3P mutation selected for the double substitution plot is indicated by the blue font at position 3 of Panel 2B. Each substitution follows the same order: the 8 non-polar residues (AFILMVWP; orange); the 7 non-polar residues (GSYCQTN; green); the 3 positively charged residues (RKH; blue); and the 2 negatively charged residues (DE; pink). The effect of deletions is shown by red bars (Δ).

At the ‘peptide maturation with substitution libraries’ step (see Fig. 1), we generated a series of iterative libraries containing all possible single, double substitutions and deletion variants of candidate binders selected in the prior two steps of the process (see Materials and Methods). As an example, LGEYH peptide selected from the 5-mer library was extended to create a XXLGEYHXX library, where X is one of the 20 natural amino acids. From the aforementioned library, the 9-mer peptide DYLGEYHGG showed the highest signal intensity was extended to a 12-mer peptide and tested for specificity as follows. First, DYLGEYHGG 9-mer peptide was further extended by two glycine amino acids at the N-terminus, GGDYLGEYHGG, and a substitution library (single/double/deletion) was generated and tested for streptavidin binding. Second, one of the top sequences in this library, FEDYLGEYHGG, was further extended on the N-terminus by a single glycine to create the 12-mer GFEDYLGEYHGG and a substitution library generated. The single substitution plot shown in Fig. 2A for this peptide validated high specificity of majority of the residues, except for glutamate at position 3, for which an E3P substitution was preferable. For this peptide the effect of E3P to the relative signal intensity was much more significant than F2L, or any other substitution. A substitution plot generated for the peptides from the same array library with a fixed proline at position 3 (double substitution plot, Fig. 2B) showed almost 2-fold improvement in overall binding signal intensity without loss of sequence specificity.

### D-amino acid peptides binding to streptavidin

The same stepwise evolution approach (Fig. 1) was followed to identify hotspot D-amino acid sequences that bind to streptavidin. Again, a 2,476,099 5-mer peptide library was synthesized with 19 of 20 D-amino acids (excluding cysteine) and fluorescence signal intensities of bound Cy5-SA across three arrays was compared to identify 114 5-mer D-peptides with the highest S/B values (Supplementary dataset Table 3). Two 5-mer hotspots, “wqeea” and “lanvd”, were selected for the extension step with all 160,000 possible combinations of D-amino acid dimers. As with the L-amino acids, the extended D-peptides showed a preference for specific sequences at both termini (Supplementary Figure 2). Consensus sequences identified with the extension libraries for each hotspot sequence are shown in the inset table in Fig. 1.

Similar to L-peptides, D-peptides with highest fluorescence intensity after extension were further matured by synthesizing series of the substitution and deletion libraries as outlined in Fig. 1. Examples of the substitution plots for D-peptides are shown in Supplementary Figures 7 and 8; sequences of the matured peptides used in the following experiments are shown in the inset table in Fig. 1.

### Cyclic L/D-amino acid peptides binding to streptavidin

To identify cyclic peptide binders to streptavidin, Cy5-SA was bound to an array library of 388,962 5-mer L/D-peptides. The pentameric peptides were composed of a 4-mer peptide synthesized with a combination of uncharged L- and D-amino acids and a γ-Glu: this residue enabled synthesis of both head-to-tail cyclic and linear peptides, respectively, for each peptide through either allyl ester or t-butyl ester C-terminal protection (see Materials and Methods for amino acid composition and cyclization protocol). The paired cyclic and linear array features were spatially positioned side-by-side to control for potential variations in cyclization yield for each peptide. The fluorescence signal intensities across three independent replicates on the same array were compared and the ratio cyclic to linear peptide of signal intensities was compared. The cyclic peptide with the highest fluorescence signal intensity was NQpW[γ-Glu], while the comparable linear peptide showed no measurable fluorescence signal intensity on the array (Supplementary Fig. 3).

### SPR analysis of peptide/streptavidin interactions

SPR analysis of peptide binding to streptavidin was performed with streptavidin immobilized on a chip and peptides in solution (see Materials and Methods). Binding curves shown in Supplementary Figure 4 are for all L-, D-, and cyclic L/D- peptides and their summarized affinity/kinetics parameters listed in Table 1. The EWVHPQFEQKAK peptide (found in this study) and Strep-tag II HPQ [used as a control^20^], demonstrated “fast on/fast off” steady-state kinetics [Supplementary Fig. 4(A,B)], with dissociation constant (K_d_) values of 5.7 µM and 49.8 µM, respectively. The L/D-cyclic peptide NQpWQ also demonstrated “fast on/fast off” kinetics, with a K_d_ value of 61.3 µM [Supplementary Fig. 4(I)]. As the array data would predict, the linear version, NH_2_-NQpWQ-COOH, exhibited no measurable binding at peptide concentrations of ≤2 mM. The GNSFDDWLASKG L-peptide demonstrated irreversible binding kinetics, making it impossible to determine a K_d_ value [Supplementary Fig. 4(C)]. The binding kinetics for the two other L-peptides and all three D-peptides showed typical association/dissociation 1:1 binding kinetics [Table 1 and Supplementary Fig. 4(D–H)]. The AFPDYLAEYHGG L-peptide had the lowest K_d_ value, 43 nM, among all peptides discovered in this work.

### Co-crystal structures of array-matured peptides with streptavidin

We determined high-resolution (between 1.05Å–1.61 Å) co-crystal structures for the 7 matured peptides listed in the inset table in Fig. 1 and for head-to-tail cyclic peptide NQpWQ to reveal the details of peptide/streptavidin interactions (Supplementary Table 1)

**Figure 3:**
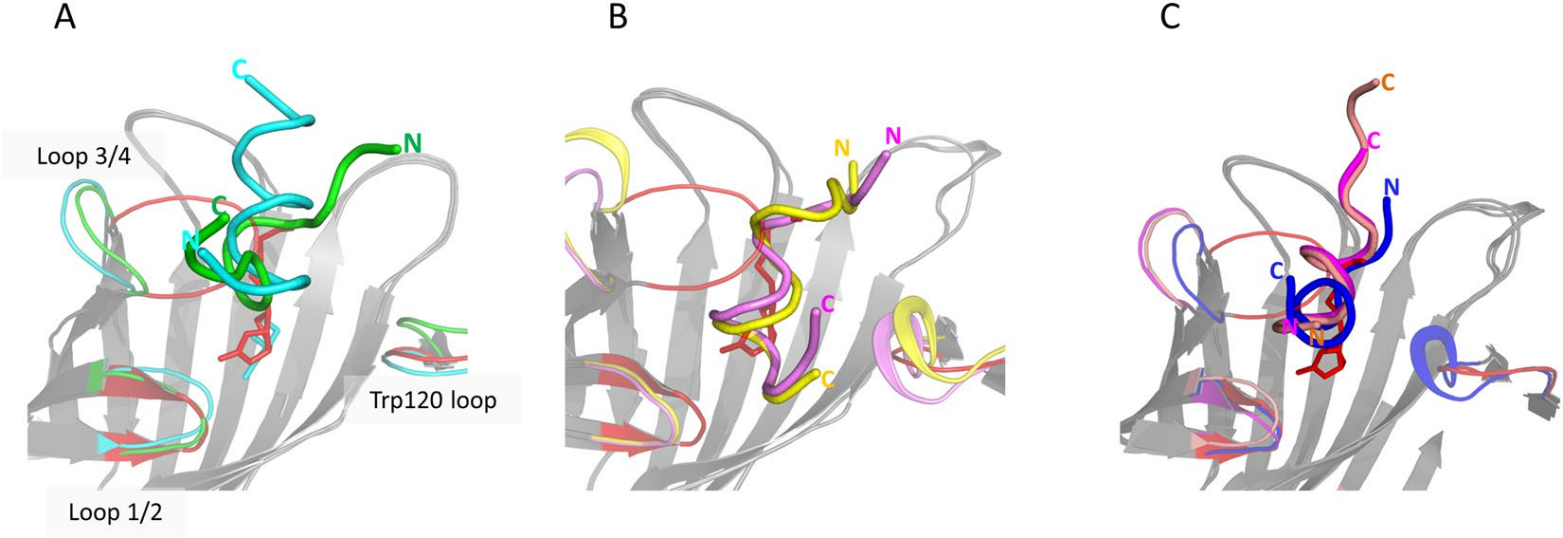
Alignment of peptide-streptavidin structures with the streptavidin-biotin complex as a reference (red). Peptides are shown as ribbons. Streptavidin molecules are shown as cartoons with structurally rigid regions colored in gray and flexible loop 1/2 (aa 22–28) and loop 3/4 (aa 42–52) of subunit A and Trp120 loop (aa 111–122) of subunit D regions in the corresponding peptide color. (**A**) EWVHPQFEQKAK (green) and GNSFDDWLASKG (cyan) peptides. (**B**) AFPDYLAEYHGG (yellow) and RDPAPAWAHGGG (violet) peptides. (**C**) GGwhdeatwkpG (salmon), Gdlwqheatwkkq (magenta), and GyGlanvdessG (blue) D-peptides. N- and C-termini are marked by N and C, respectively. All structures are viewed from the same perspective.

All peptides bind within or near the biotin binding pocket of streptavidin formed by two surface loops [1/2 (amino acids 22–28) and 3/4 (amino acids 42–52)], and by antiparallel β-sheets involved in an extensive polar interaction network, in which residues Ser88 and Thr90 of β-strand 6 play a major role (Fig. 3). The surface loops of streptavidin are flexible: upon biotin binding they undergo a conformational change to form a closed conformation^21^, but to accommodate the peptide ligands, loop 3/4 is dislocated by 13–16 Å from the biotin closed form to adopt a well-defined peptide-specific structure. The loop region around Trp120 of the neighboring subunit provides additional contacts within the streptavidin tetramer. With the exception of these flexible loops, streptavidin has a rigid structure, with a root mean square deviation (rmsd) of ~0.5 Å for a superposition of all atoms in the co-crystal structures. The Gdlwqheatwkkq, GGwhdeatwkpG and GNSFDDWLASKG peptides bind with the same N-terminus to C-terminus directionality, opposite to the binding orientation of all other peptides.

All contacts between peptides and streptavidin at the distance of 4 Å and the hydrogen bond and polar interactions of biotin with streptavidin are listed in Supplementary Table 4.

### Detailed analysis of correlation between array and crystallography data

**Figure 4:**
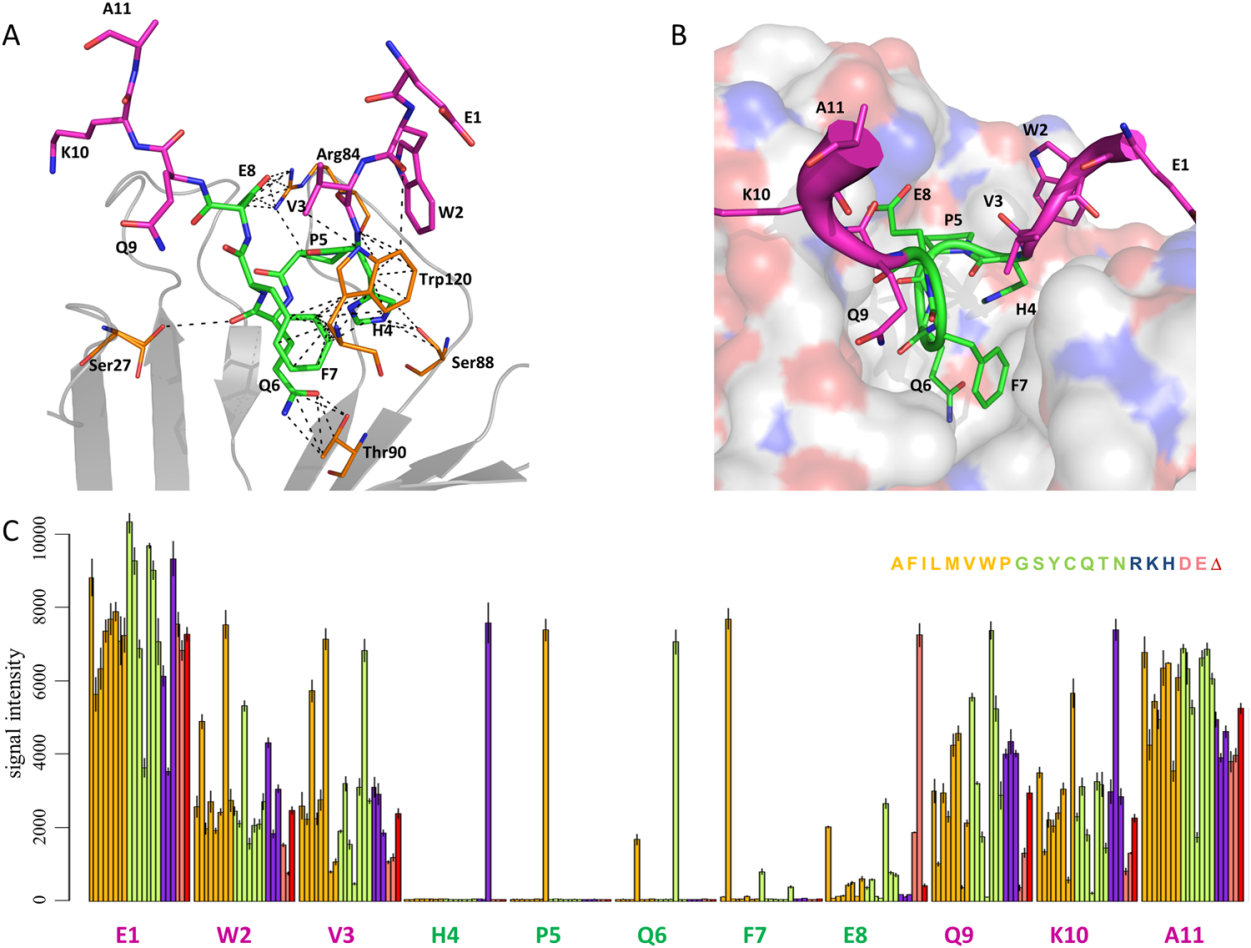
Co-crystal structure with streptavidin and specificity plot for the EWVHPQFEQKAK peptide. (**A**) Key interactions within 4 Å (dashed lines) between the peptide (green and purple, indicated by the single-letter amino acid code) and streptavidin (orange, indicated by the three-letter amino acid code). (**B**) A view into the biotin-binding pocket of streptavidin (shown as a surface) with bound peptide (main chain shown in B-factor putty representation in green and purple). (**C**) Substitution plot showing signal intensity for 20 amino acid substitutions and a deletion for each peptide position. The color coding scheme for the bars representing signal intensity is the same as in Fig. 2. Key HPQFE residues are shown in green; other amino acids are shown in purple.

*L-*peptides. Binding conformation of EWVHPQFEQKAK peptide closely resembles previously published HPQ peptide-streptavidin structures^22^. The HPQFE amino acids at positions 4–8, occupy the biotin-binding pocket of streptavidin and adopt a rigid conformation, whereas the N- and C-terminal amino acids are exposed to the solvent and show significant degree of freedom in the crystal structure [Fig. 4(B)]. The His4 and Gln6 sidechains of the HPQ motif form hydrogen bonds with residues Ser88 and Thr90 of streptavidin. Pro5 is crucial for positioning the Gln6 sidechain within hydrogen bonding distance of Thr90. The sidechain of Phe7 is involved in critical π-π stacking against Trp120 from a neighboring streptavidin subunit. Further, at position 8, the negatively charged, long sidechain of Glu8 is important for charge-charge interactions with Arg84 [Fig. 4(A)]. In the substitution plot for the EWVHPQFEQKAK peptide [Fig. 4(C)], the most specific region is the HPQFE middle portion, which forms a “specificity valley”, surrounded by much less specific N- and C- terminal regions [Fig. 4(C)].

**Figure 5:**
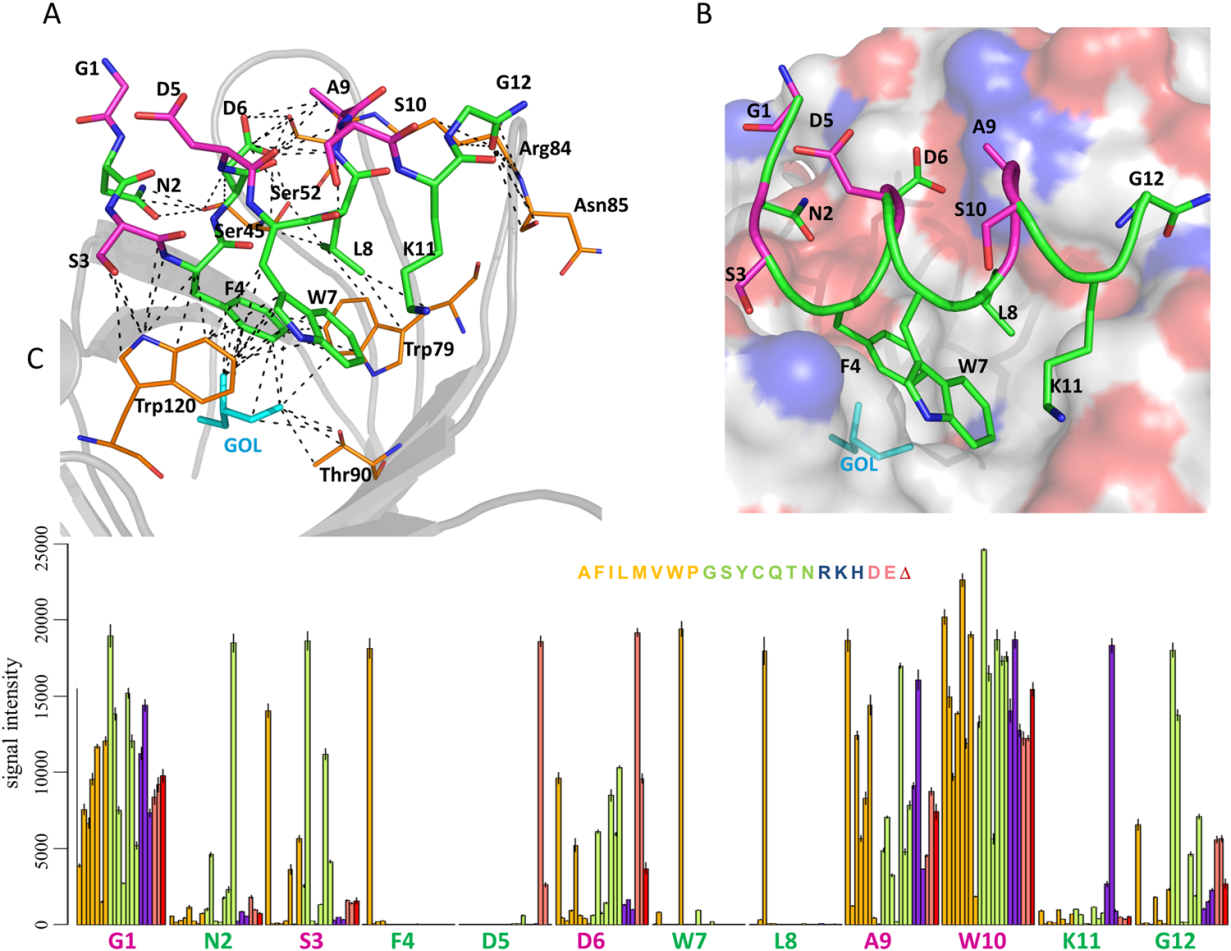
Co-crystal structure with streptavidin for the GNSFDDWLASKG peptide and specificity plot for its S10W variant. (**A**) Key interactions within 4 Å (dashed lines) between the peptide (green and purple, indicated by the single-letter amino acid code) and streptavidin (orange, indicated by the three-letter amino acid code). (**B**) A view into the biotin-binding pocket of streptavidin (shown as a surface) with bound peptide (main chain and side chains shown as ribbons in green and purple). (**C**) Substitution plot showing signal intensity bars for 20 amino acid substitutions and a deletion for each peptide position. The color coding scheme for the bars representing signal intensity is the same as in Fig. 2. Key specific amino acids are shown in green; the other amino acids are shown in purple.

In contrast to the EWVHPQFEQKAK peptide, the GNSFDDWLASKG peptide forms an α-helix and is located outside the biotin-binding pocket which is occupied by a glycerol molecule [Fig. 5(A,B)], so that the peptide does not directly participate in polar interactions with Ser88 and Thr90. Instead, Asn2 forms a hydrogen bond with Ser45; the backbone NH of the C-terminal Gly12 forms a hydrogen bond with Asn85. The negatively charged sidechain of Asp5 forms a salt-bridge with Arg84 and contributes to a network of polar interactions with Ser52 and Ser45. Further, Phe4, Trp7, and Leu8 are involved in critical van der Waals interactions with Trp79 and Trp120 from a neighboring streptavidin subunit [Fig. 5(A)].

All mentioned peptide residues—Asn2, Phe4, Asp5, Trp7, Leu8, and Gly12—demonstrate high specificity in the substitution plot [Fig. 5(C)]. For example, the substitution plot reveals a clear preference for Asp over Glu at position 5, which can be explained by the multiple polar interactions that the short Asp5 sidechain—but not the longer Glu sidechain—can accommodate. Because GNSFDDWLASKG forms an α-helix, a repeating pattern of conserved residues alternates with non-conserved positions in steps of 2–3 amino acids.

Strong correlation between specificity in the substitution plots and co-crystal data was also observed for the AFPDYLAEYHGG peptide (the highest affinity for streptavidin in SPR measurements) and the RDPAPAWAHGGG peptide (the longest stretch of highly specific amino acids between positions 2 and 11) (Supplementary Fig. 5 and Supplementary Fig. 6, respectively).

*D-*peptides. All D-peptides fold into a left-handed α-helix with one turn for GyGlanvdessG and two turns each for the Gdlwqheatwkkq and GGwhdeatwkpG peptides. Gdlwqheatwkkq and GGwhdeatwkpG share high sequence homology; further, their conformation and binding mode are highly similar [Fig. 3(C) and Supplementary Fig. 7(A)]. In the common “wxxea” core of these two peptides, the D-Trp forms polar contacts with Asp128 and contributes an edge-to-face interaction with Trp108, whereas the D-Glu shares a salt bridge with Arg84. D-Ala is critical to the core motif because its short sidechain perfectly accommodates the limited space within the pocket. The positions “xx” of the “wxxea” core face the solvent and therefore are less specific [Supplementary Fig. 7(B,C)]. For the GyGlanvdessG peptide, the co-crystal structure supports the importance of residues 3–10 as indicated by the substitution plot [Supplementary Fig. 8(A)], while the substitution-tolerant N- and C-terminal ends of the peptide are disordered in the structure [Supplementary Fig. 8(B)].

**Figure 6:**
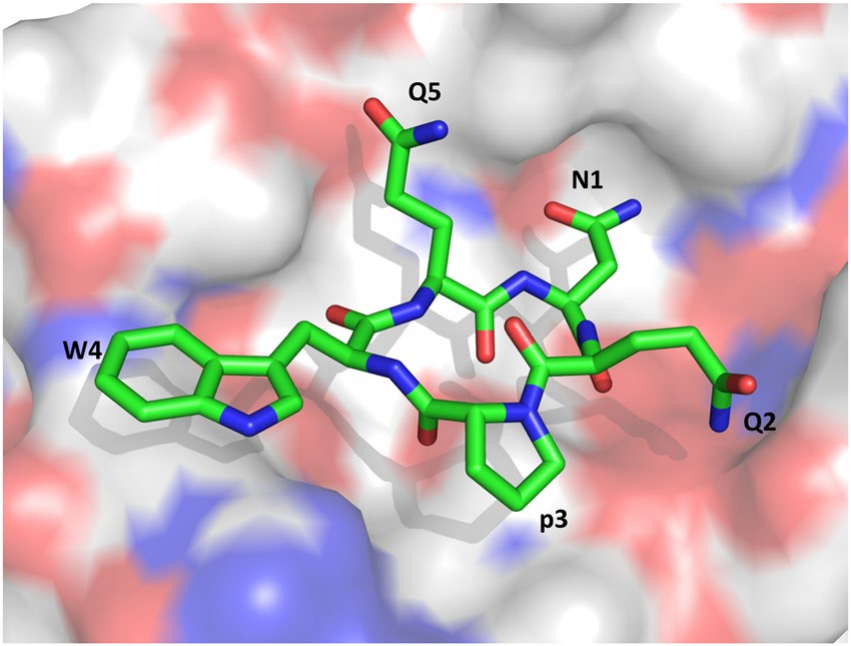
View of the binding site of streptavidin (depicted as a surface representation for the streptavidin subunit) with the head-to-tail cyclic NQpWQ peptide (shown as sticks).

*Cyclic* peptide. The peptide adopts a flat disc conformation with all amino acid side chains (except Gln5) localized in the same plane (Fig. 6). Asn1 and Gln5 engage in polar interactions with Thr90 and Ser88, respectively. Further, the backbone carbonyl oxygen of Asn1 is hydrogen-bonded to Ser27 and Tyr43.

## Discussion

To demonstrate the feasibility of stepwise evolution and rational peptide array design (Fig. 1), we selected streptavidin as a target molecule. For almost three decades, streptavidin has been a classic model for evaluating various screening techniques, including phage display, mRNA display, and combinatorial peptide bead libraries^13–18^. The most frequently identified peptide sequences in the majority of these studies contain the same consensus HPQ(M) motif^23,24^, with very few examples not belonging to this family^25^. The importance of the HPQ motif has been demonstrated by multiple co-crystal structures^20,22,26,27^.

Indeed, we found that HPQ(M) was the predominant motif among the top 5-mer L-peptides discovered in the initial screen of the 5-mer library. When we examined the top 1,100 sequences that bound to streptavidin, only 81 contained “secondary” binding motifs, rather than the HPQ(M) motif. Here, we took advantage of the “spatially addressable features” of our technology: we could assess the relative binding affinity of not only the most dominant peptide families, but also peptide families exhibiting weaker, but nonetheless detectable, binding.

From these “secondary” peptide families identified in the initial screen, we chose three 5-mer “hot spot” sequences: LAEYH, PAWAH, and FDEWL. To the best of our knowledge, none of these sequences have been reported previously. We used a series of array peptide libraries rationally designed around each of these sequences to successfully evolve the three sequences to 12-mer L-peptides. One L-peptide, AFPDYLAEYHGG, showed a K_d_ of 43 nM—a 100-fold improvement over the HPQ peptide also developed in this study, and 1,000-fold greater than Strep-tag II HPQ (Table 1). Finding a 12-mer peptide using current random library selection technologies would theoretically require the initial random library to possess diversity greater than 4 × 10^15^; such a library would be several orders of magnitude larger than any library of practical size.

**Table 1:** SPR measured equilibrium dissociation constant (K_d_), association rate constant (k_a_), and dissociation rate constant (k_d_) for streptavidin’s interaction with matured L- or D-peptides (see inset table in Fig. 1), L/D-cyclic peptide, and SAWSHPQFEK (Strep-tagII) peptide^20^.

Peptide	K_d_, µM	k_a_, 1/Ms	k_d_, 1/s
**L-peptides**			
NH_2_-SAWSHPQFEK-COOH (Strep-tag II)	49.8 ± 2.4		
NH_2_-EWVHPQFEQKAK-amide	5.7 ± 0.29		
NH_2_-GNSFDDWLASKG-amide	irreversible binding		
NH_2_-AFPDYLAEYHGG-amide	0.043	(3.0 ± 0.11) 10^4^	(1.3 ± 0.029) 10^−3^
NH_2_-RDPAPAWAHGGG-amide	4.7	(1.5 ± 0.02) 10^4^	(7.0 ± 0.026) 10^−2^
**D-peptides**			
NH_2_-GyGlanvdessG-amide	2.87	(2.48 ± 0.02) 10^4^	(7.1 ± 0.039) 10^−2^
NH_2_-Gdlwqheatwkkq-amide	1.84	(0.65 ± 0.02) 10^4^	(1.2 ± 0.011) 10^−2^
NH_2_-GGwhdeatwkpG-amide	0.47	(8.1 ± 0.02) 10^4^	(3.8 ± 0.016) 10^−2^
**L/D-cyclic peptide**			
NQpWQ (head-to-tail cyclic)	61.3		
NH_2_-NQpWQ-COOH	no measureable binding (>2000)		

To explain why “secondary” binders that exhibit higher affinity to streptavidin than the commonly reported HPQ-motif binders have not been previously selected in random libraries, we assumed that the HPQ(M) motif contributes most of the binding energy, whereas the contribution of the flanking sequences is relatively minor and multiple flanking sequences are acceptable. Thus, HPQ(M)-peptides can *en masse* outcompete alternative candidates that have longer core sequences and that might be present in smaller copy numbers in the initial rounds of random library selection. We believe that the “winner takes all” bias could be a general phenomenon inherent to all display technologies, and thus limits their utility.

The benefits of employing a systematic screening approach rather than random library selection have been demonstrated^6,28^, but faces the challenge of synthesizing focused, yet adaptable libraries. The array synthesis technology described here has made stepwise evolution practical and possible for three reasons: (1) the high density of the peptide arrays enabled placement of nearly all possible 5-mer peptides on a single array, even for an initial screen; (2) the high sensitivity enables detection of even low-affinity binding events; and (3) the efficient array design and synthesis process enabled rapid completion of multiple rounds of binder evolution.

Notably, rational peptide array design enables not only identification of high-affinity peptide binders but also assessment of the effects of individual amino acid substitutions at each position in a single experiment, as clearly demonstrated by the single/double amino acid substitution plots (Fig. 2). Unlike the well-known alanine scan, this method both compares the binding of thousands of related peptide variants and identifies critical binding residues.

One concern is that the substitution plots might reflect artifacts of array-synthesized peptides and/or the surface microenvironment rather than *bona fide* peptide interactions with the target. Although these effects cannot be completely excluded, comparing the substitution plots with the co-crystal structures for each peptide revealed an excellent correlation between the binding specificity of the preferred amino acids and their relative contribution to the interactions with streptavidin in the co-crystal structure. The highly specific amino acids identified by substitution analysis face the streptavidin binding pocket and participate in an elaborate network of inter-/intra-molecular interactions (Figs 4, 5, and Supplementary Figs 5–8). Similarly, amino acids that are exposed to solvent and do not make contacts with streptavidin represent non-specific peptide positions.

All eight L- and D-peptides discovered in this work (Table 1) bind to the same pocket of streptavidin in distinctive binding modes suggesting (1) the rich malleability of L- and D-peptides and (2) the ability of streptavidin itself to adjust the flexible loop to accommodate multiple peptides with diverse sequences and conformations(Fig. 3). Interestingly, five of eight peptides found here adopt an α-helical conformation, underscoring the importance of intra-peptide interactions for peptide/target stability. The observation that highly diverse peptides can bind to the same pocket is likely not unique to streptavidin; we expect that the described approach will enable the discovery of multiple peptide binders for other targets (manuscript in preparation).

Our combined stepwise evolution and rational peptide array design approach could potentially advance drug discovery by designing peptide libraries that incorporate additional modifications (e.g., β-amino acids, N-methyl amino acids, and peptoids) to expand the libraries’ physicochemical and conformational diversity. These libraries, together with array-based assays, could be used to select for binding affinity and to assess proteolytic stability and cell permeability. Ultimately, this approach could both greatly shorten the time needed for lead molecule discovery and enable identification of compounds with integrated drug-like properties including oral availability, good pharmacokinetics, and low toxicity in a single screen.

## Methods

Methods and the associated references are available in the online version of the paper.

### Peptide array synthesis

Peptide synthesis was accomplished through light-directed array synthesis in a Roche NimbleGen Maskless Array Synthesizer (MAS) using an amino-functionalized substrate as previously reported^29^.

The combined cyclic and linear peptide libraries were synthesized starting with either the allyl ester (OAll) or t-butyl ester, respectively, of N-(2-nitrophenyl)propoxycarbonyl (NPPOC)-protected glutamate (γ-Glu) linked to the array surface through the carboxylic acid side chain. To cyclize the peptides prior to side chain deprotection, the array was first treated with tetrakis(triphenylphosphine)palladium(0) (2 mM) in THF for 3 h at room temperature to remove the OAll protecting group from the C-terminus of the peptide library. To remove residual palladium from the array, the slide was washed with 5% N,N-diisopropylethylamine (DIPEA) and 5% sodium diethyldithiocarbamate in DMF for 5 min. After a 1-min wash with water, the slide was spun to dryness before cyclization. The array was then cyclized by coupling the N- to the C-terminus using a standard coupling procedure: (1) the slide was treated with activator (HOBT and HBTU, 20 mM each) and base (DIPEA, 2 M) for 3 h at room temperature; (2) the cyclized array was then side-chain deprotected in TFA (47.5 mL), triisopropylsilane (0.25 mL), and water (2.25 mL) for 30 min at room temperature; (3) the slide was then washed: (a) twice in methanol for 30 sec, (b) 4 times in water for 10 sec, (c) TBS with 0.05% tween-20 for 2 min, and then (d) TBS for 1 min; (5) finally, the slide was spun to dryness.

### Array design

#### HotSpot Discovery Arrays

The comprehensive peptide library included 2,476,099 5-mer peptides synthesized in a single copy with 19 of 20 L- or D-amino acids (excluding cysteine). Libraries were flanked on both N- and C-termini by linkers of 1 or 3 amino acids using a 3:1 glycine-serine mixture.

#### HotSpot Extension Arrays

Extension libraries were designed using a fixed-core sequence extended at both the N- and C-termini with all possible 20 L- or D-amino acid dimers. Each library included 160,000 unique peptides synthesized in five replicates. Each array accommodated up to three independent extension libraries.

#### Substitution Arrays

Substitution libraries were designed by introducing all possible single- and double-amino-acid substitutions and single-amino-acid deletions for a specific sequence using all 20 L- or D-amino acids. Each library was synthesized in five to seven replicates. Each array accommodated up to 12 independent substitution libraries.

#### Cyclic Discovery Arrays

All peptides in the library were 5-mers in the format XXXX[γ-Glu], where XXXX is a combination of all possible 4-mer amino acids from a subset of L- and D-amino acids, and γ-Glu is a L-glutamate protected on the C-terminus with either an allyl ester or a t-butyl ester, to generate cyclic or linear features, respectively, as described above. The L-amino acids included in this design were Ala, Asn, Gln, Gly, Ile, Leu, Phe, Pro, Ser, Thr, Trp, Tyr, and Val; the D-amino acids were Ala, Asn, Leu, Phe, Pro, Ser, Trp, and Tyr.

### Streptavidin binding on array

Cy5™-streptavidin (Cy5-SA) was purchased from GE Healthcare (Little Chalfont, UK). Freshly deprotected arrays were used in each experiment. Streptavidin binding to all arrays was performed with 0.5 µg/ml Cy5-SA either in binding buffer containing 10 mM Tris-HCl (pH 7.4), 1% alkali-soluble casein (EMD Millipore), 0.05% Tween-20 or in 10 mM Tris-HCl (pH 7.4), 4% BSA (Roche, Basel, Switzerland), 0.05% Tween-20 in a 30 mL PAP Jar container (Evergreen Scientific, Vernon, CA) overnight at 4° C. After incubation, arrays were washed in 20 mM Tris-HCl (pH 7.8), 0.2 M NaCl, 1% SDS or 1X TBS (pH 7.4) for 30 sec followed by a 1 min wash in water, and then dried by spinning in a microcentrifuge equipped with an array holder.

### Data analysis

Cy5 fluorescence intensity of the arrays was measured with an MS200 scanner (Roche NimbleGen, Madison, WI) at resolution 2 µm, wavelength 635 nm, gain 25%, and laser intensity 100%. Cy5 signal intensities were extracted using Image Extraction Software (Roche NimbleGen). Data pre-processing, normalization, and statistical tests were performed using the language R. Data visualization and analysis was performed with the Spotfire 6.5.0 (Tibco, Boston, MA) software platform. Distance analysis and principle component analysis of distance matrices were performed with the R package PEPLIB^19^.

### Peptide synthesis

All peptides were provided at 98–99% purity and used as received. Strep-tag II peptide, NH_2_-SAWSHPQFEK-COOH (Strep-tag II HPQ), was purchased from IBA GmbH (Goettingen, Germany). The cyclic (head-to-tail) and linear versions of peptide NQpWQ were purchased from GenScript (Piscataway, NJ). All other peptides were synthesized by either the University of Wisconsin Biotechnology Center (Madison, WI) or by Peptide 2.0 (Chantilly, VA).

### SPR experiments

Surface Plasmon Resonance (SPR) experiments were performed using a Biacore X100 instrument (GE Healthcare). 60 µl of 100 µg/ml streptavidin in 10 mM Na-acetate (pH 5.0) was immobilized to flow cell 2 (Fc2) of a sensor chip CM5 (GE Healthcare) using the Amine Coupling Kit (GE Healthcare) at 20 °C for 6 min. Peptide stock solutions were prepared at 5 or 10 mM in H_2_O and diluted in HBS-EP+ (GE Healthcare) buffer. Peptide binding was performed in a multiple kinetics mode using HBS-EP+ as a running buffer and 0.2 M NaCl, 10 mM NaOH, or 10 mM HCl-glycine (pH 1.7) as the regeneration buffer. Binding kinetics parameters were calculated using Biacore X100 software.

### Crystallization and data collection

Crystallization screening for streptavidin (Roche Diagnostics, Risch-Rotkreuz, Switzerland) and peptides was performed at 21 °C in vapor diffusion sitting-drop experiments at streptavidin concentrations of 20–30 mg/ml. Crystals were obtained by mixing 0.14 µL protein with 0.06 µL of screening solution (Procomplex, Qiagen, Hilden, Germany). Details regarding protein-peptide incubation ratios and times, concentrations and crystallization solutions are summarized in Supplementary Table 1. Various crystal forms were found in each peptide co-crystallization experiment. The first crystals appeared within minutes, mainly in polyethylene glycol-containing solutions, and grew to their final size within 3 days after setup. Crystals could be directly harvested out of the screening plate without any further optimization steps because crystal size and quality were sufficient for data collection. For cryoprotection, crystals were transferred into crystallization solution supplemented with 20% glycerol. Diffraction data were collected at the Swiss Light Source (Villigen, Switzerland) on beamline X10SA using a Pilatus 6 M detector.

### Structure determination and refinement

Data were processed with Extended Data Services^30^ and scaled using SADABS x-ray diffraction (Bruker, Billerica, MA). Structures were determined by molecular replacement with PHASER^31^ using the apo-streptavidin coordinates of Protein Data Bank (PDB) entry 3RY1. With programs from the CCP4 suite^32^ and BUSTER^33^, the coordinates obtained by molecular replacement were subsequently refined by rigid-body and positional refinement (Supplementary Table 1). Manual rebuilding of the protein was achieved using model-building software (COOT^34^). The difference electron density was used to rebuild the loop areas and to place the peptides. Distance calculations and analysis of contacts between streptavidin and the peptides were conducted in COOT and with the molecular modeling/simulation program MOE^35^. Images were produced with the structural visualization program PYMOL^36^.

## Electronic supplementary material

Supplementary Information

Table 2A

Table 2B

Table 3
