# Supplementary material for: Stepwise Evolution Improves Identification of Diverse Peptides Binding to a Protein Target
Source: Sci Rep. 2017 Sep 21;7:12116. doi: 10.1038/s41598-017-12440-1 (PMC5608804; doi:10.1038/s41598-017-12440-1)
Supplement: Supplementary file 1 — Supplementary Information [file 41598_2017_12440_MOESM1_ESM.pdf]

STEPWISE EVOLUTION IMPROVES IDENTIFICATION OF DIVERSE PEPTIDES BINDING TO A PROTEIN TARGET

Victor Lyamichev[1], Lauren Goodrich[1], Eric Sullivan[1], Ryan Bannen[1], Joerg Benz[2], Thomas Albert[1], Jigar Patel[1]

[1]Roche Madison, Madison, WI 53719
[2]Roche Pharmaceutical Research and Early Development, Therapeutic Modalities, Roche Innovation Center Basel, Grenzacherstrasse, 4070 Basel, Switzerland

Supplementary Table 1. Crystallization of streptavidin and peptides, including data collection and refinement statistics.

**Peptide sequence**	EWVHPQFEQKAK	GNSFDDWLASKG	AFPDYLAEYHGG	RDPAPAWAHGGG	GyGlanvdessG	Gdlwqheatwkkq	GGwhdeatwkpG	NQpWQ
Data collection								
**Peptide-protein complex formation**	Peptide stock:<br>50mM in water<br>Incubate 3h at RT in 1:2 molar access	Peptide stock:<br>10mM in water<br>Incubate 3h at RT in 1:2 molar access	Peptide stock:<br>50mM in Hepes 7.0<br>Incubate 3h at RT in 1:3 molar access	Peptide stock:<br>50mM in water<br>Incubate 3h at RT in 1:2 molar access	Peptide stock:<br>50mM in DMSO<br>Incubate 4h at RT in 1:3 molar access	Peptide stock:<br>10mM in water<br>Incubate 3h at RT in 1:3 molar access	Peptide stock:<br>10mM in water<br>Incubate 3h at RT in 1:3 molar access	Peptide stock:<br>5mM in water<br>Incubate ON at RT in 1:3 molar ratio
**Crystallization condition Procomplex (Qiagen)**	0.1M Magnesium acetate<br>0.1M Sodium cacodylate pH 6.5<br>15% PEG6000	0.1M Potassium chloride<br>0.1M HEPES pH 7.5<br>15% PEG6000	0.1M Magnesium chloride<br>0.1M Sodium citrate pH 5<br>15% PEG4000	0.2M Ammonium acetate<br>0.1M Sodium acetate pH 4<br>15% PEG4000	0.1M Sodium citrate pH 5.6<br>20% PEG4000<br>20% Isopropanol	0.1M Sodium citrate pH 4.5<br>20% PEG4000	0.1M Magnesium acetate<br>0.1M MOPS pH 7.5<br>12% PEG8000	0.1M Sodium citrate pH 5.5<br>15% PEG6000
**Peptide occupancy**	All 8 sites	All 8 sites	All 4 sites	3 sites occupied/1 with unidentified mol.	3 of 4	1 of 1	2 of 2	All 12 sites
**Resolution (Å)**	80.1-1.12	85.5-1.27	75.1-1.03	57.8-1.10	57.09-1.05	47.7-1.61	62.3-1.10	48.72-1.50
**Wavelength (Å)**	0.99992	0.70000	0.77491	0.99992	0.70002	0.70001	0.99999	0.99999
**Space Group**	P1	P1	P1 21 1	P1 21 1	P1 21 1	P42 21 2	C2 2 21	P21 21 21
**Cell dimensions**<br>**a,b,c (Å)**<br>**α,β,γ (°)**	57.1, 64.8, 96.3<br>96.3, 97.1, 84.1	58.3, 58.3, 88.3<br>104.4, 91.0, 88.4	51.3, 65.8, 78.1<br>90, 103.7, 90	46.8, 84.7, 58.5<br>90, 99.4, 90	46.7, 84.9, 57.8<br>90, 99.1, 90	57.8, 57.8, 84.4<br>90, 90, 90	81.9, 95.7, 82.1<br>90, 90 ,90	67.7,115.3,210.4<br>90, 90 ,90
**No. Molecules in AU**	8	8	4	4	4	1	2	12
**Total reflections**	642986	473781	458324	331071	396326	35419	249576	693361
**Unique reflections**	403025	273857	235380	167056	195681	18228	122566	261661
**Multiplicity ()**	1.66 (1.53)	1.74 (1.70)	3.38 (3.33)	3.17 (2.75)	3.43 (3.40)	12.48 (12.93)	6.20 (5.29)	6.58(5.77)
**Completeness (%)**	90.4 (86.1)	97.0 (95.7)	99.5 (99.7)	96.7 (85.3)	99.6 (99.7)	100 (100)	99.2 (96.8)	99.7 (98.4)
**Mean I/σ(I)**	10.03 (0.94)	7.71 (0.90)	9.44 (1.14)	15.2 (4.26)	11.4 (1.11)	14.94 (0.86)	17.4 (4.01)	13.3 (1.49)
**Rsym**	0.031 (0.54)	0.072 (0.67)	0.045 (0.65)	0.031 (0.28)	0.036 (0.79)	0.057 (0.95)	0.048 (0.40)	0.079 (0.677)
**CC1/2**	99.9 (36.4)	99.8 (37.8)	99.9 (36.7)	99.9 (88.8)	100 (38.8)	100 (34.1)	100 (79.2)	99.9 (44.7)
Refinement								
**Rcryst**	16.1 (34.2)	18.4 (43.0)	14.1 (32.8)	13.5 (18.4)	17.3 (33.5)	20.2 (42.2)	13.9 (32.3)	
**Rfree**	18.6 (36.6)	20.7 (44.3)	15.7 (32.6)	15.2 (20.1)	19.8 (34.5)	24.1 (38.9)	15.4 (32.3)	19.1 (27.8)
**R.m.s. deviations**<br>**Bond lengths (A)**<br>**Bond angles (°)**	0.013<br>1.55	0.012<br>1.42	0.007<br>1.37	0.008<br>1.35	0.010<br>1.47	0.010<br>1.56	0.007<br>1.41	0.010<br>1.10
**Ramachandran (%)**<br>**Favored, allowed, outliers**	97.8, 2.2,0	97.4, 2.6, 0	96.3, 3.7, 0	97.7, 2.1, 0.2	96.2, 2.3, 1.4	95.1, 4.1, 0.8	92.9, 5, 2.1	96.6,2.6,0.8
**No. atoms**<br>**Protein**<br>**Peptide**<br>**Water**	9634<br>7572<br>792<br>1270	9317<br>7415<br>732<br>1108	5153<br>3799<br>384<br>964	4771<br>3854<br>243<br>637	4571<br>3707<br>167<br>689	1059<br>907<br>85<br>67	2502<br>1899<br>180<br>413	13995<br>11367<br>476<br>2098
***B*-factors**<br>**Protein**<br>**Peptide**<br>**Water**	14.21<br>20.83<br>29.21	15.46<br>19.41<br>27.14	9.91<br>9.17<br>27.45	10.18<br>12.14<br>22.71	8.81<br>11.08<br>22.95	28.89<br>34.56<br>35.17	7.14<br>8.81<br>22.27	21.60<br>22.96<br>35.83
**PDB code**	5N7X	5N89	5N8B	5N8E	5N8S	5N8T	5N8W	5N99

Supplementary Table 4A. Polar interactions of biotin with streptavidin (1SWE).

Biotin	Streptavidin amino acids (single-letter amino acid code)
	N23, S27, Y43, S45, N49, S88, D128, W120 (trp120)

Supplementary Table 4B. Interactions at 4.0 Å distance between peptide binders used for crystallography study (Fig. 1) and streptavidin:

EWVHPQFEQKA peptide (5N7X)

Peptide	streptavidin
E1	N118 (trp120), K121 (trp120)
W2	W120 (trp120)
V3	W120 (trp120)
H4	W79, S88, L110, W120 (trp120)
P5	S45, Y54, R84, A86
Q6	S27, W79, T90, W92, W108, L110
F7	L25, W108, L110, W120 (trp120)
E8	S45, A46, V47, S52, R84
Q9	L25, A46, V47
K10	A46, V47
A11	none

GNSFDDWLASKGG peptide (5N89)

Peptide	streptavidin
G1	none
N2	L25, S45, A46
S3	A117 (trp120), N118 (trp120 loop), W120 (trp120 loop)
F4	W79, L110, L124, W120 (trp120 loop)
D5	S45, A46, S52, R84
D6	none
W7	S88, W120 (trp120 loop)
L8	S45, Y54, W79, R84, A86
A9	R84
S10	none
K11	A86
G12	R84, N85
Glycerol/streptavidin	N23, L25, Y43, W79, T90, W92, L110, W108, L110, D128
Glycerol/peptide	F4

AFPDYLAEYHGG peptide (5N8B)

Peptide	streptavidin
A1	A86, H87, S88, S112
F2	L110, S112, S122, L124
P3	A86, W120 (trp120 loop)
D4	N118 (trp120 loop), K121 (trp120 loop)
Y5	Y54, R84, A86
L6	W79, S88, W120 (trp120 loop)
A7	W120 (trp120 loop)
E8	N23, L25, S27, S45, A46

Y9	S27, Y43, E44, S45, S52, Y54, W79, R84
H10	W79, T90, W108, L110, H127, D128, W120 (trp120 loop)
G11	N23, L25, D128
G12	L25, A117 (trp120 loop), W120 (trp120 loop)

RDPAPAWAHGGG peptide (5N8E)

Peptide	streptavidin
R1	K121 (Trp120 loop)
D2	W120 (trp120 loop), K121 (trp120 loop)
P3	A86, S88, S112, L124, W120 (trp120 loop)
A4	W79, S88, L110, W120 (trp120 loop)
P5	W79
A6	W120 (trp120 loop)
W7	N23, L25, S27, Y43, E44, S45, S52
A8	Y43, W79, W92
H9	W79, T90, W108, L110, W120 (trp120 loop)
G10	N23, L25, D128
G11	L25, A117 (trp120 loop), W120 (trp120 loop)
G12	A117 (trp120 loop), W120 (trp120 loop)

Gdlwqheatwkkq peptide (5N8T)

Peptide	streptavidin
l3	L25, S27, Y43
w4	N23, S27, Y43, W79, T90, W92, W108
q5	W108
h6	none
e7	Y43, S52, Y54, W79, A86
a8	W79, A86, S88, L110
t9	A86
w10	R84, N85, A86
k11	none

GGwhdeatwkpG peptide (5N8W)

Peptide	streptavidin
G1	L25
G2	L25, G26, S27, Y43
w3	S27, Y43, W79, T90, W92, W108, D128
h4	none
d5	none
e6	Y43, S52, Y54, W79, R84
a7	W79, A86, S88, L110

Supplementary Figure 1. Principal component analysis (PCA) for 81 'non-HPQ' sequences selected from L-amino acid 5-mer library (Supplementary Table 2B). Sequences selected for the extension step are highlighted in green color.

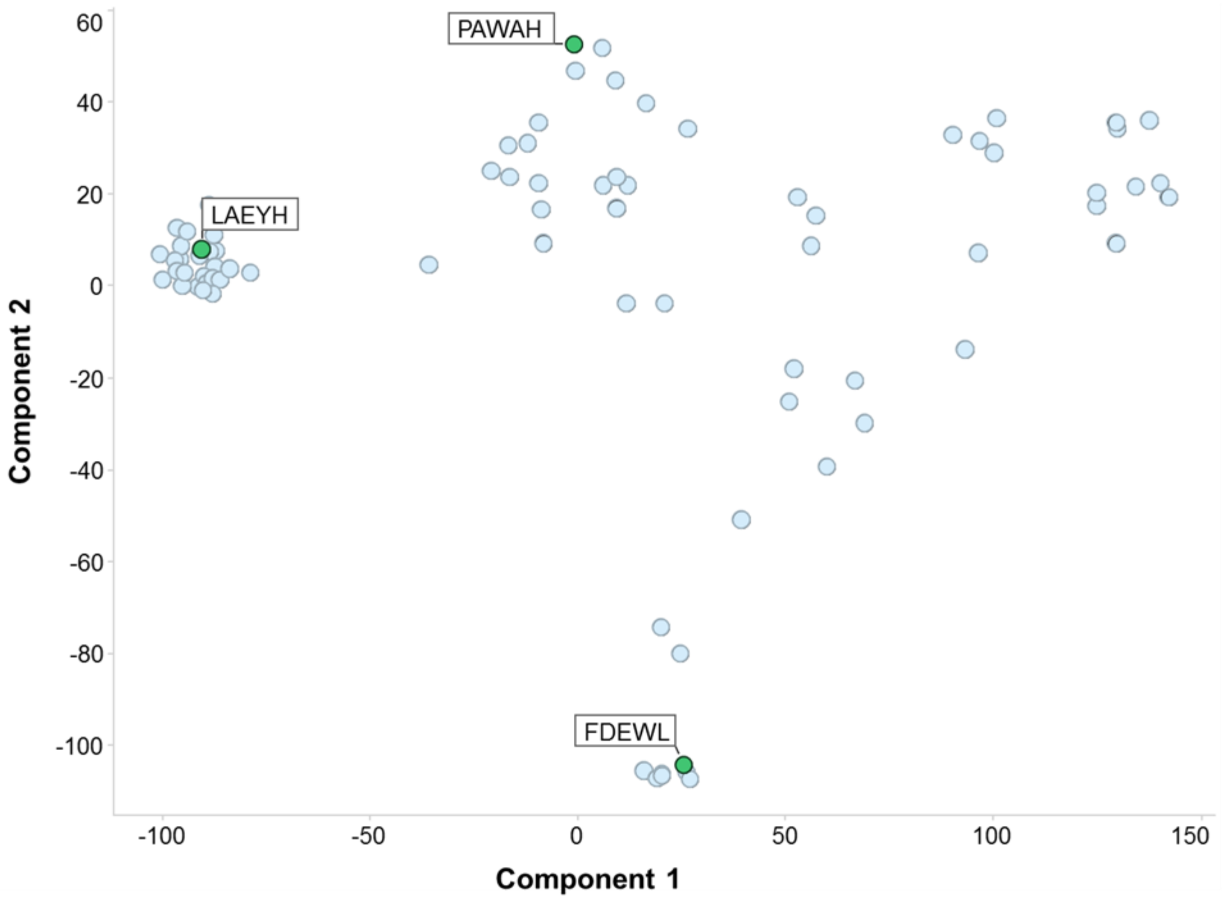

Supplementary Figure 2. Logo plots created for 15 sequences with the highest streptavidin binding signal selected from 'HPQ', 'FDEWL', 'LAEYH', 'PAWAH' L-amino acid and 'lanvd', 'wqeea' D-amino acid extension libraries.

'HPQ' extension library.

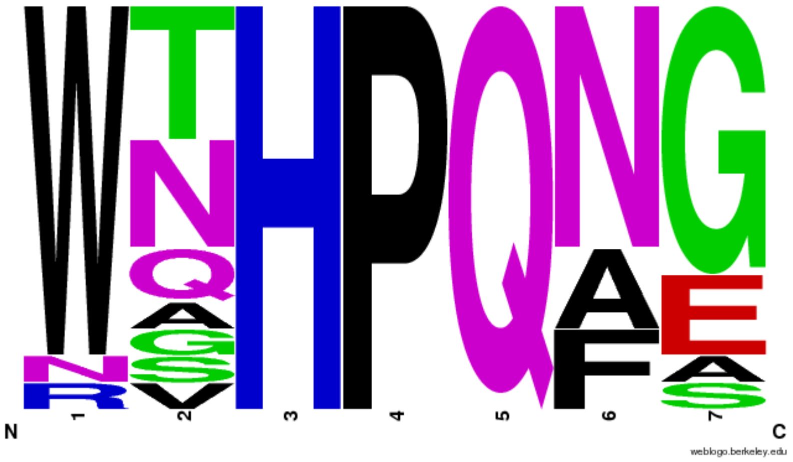

'FDEWL' extension library

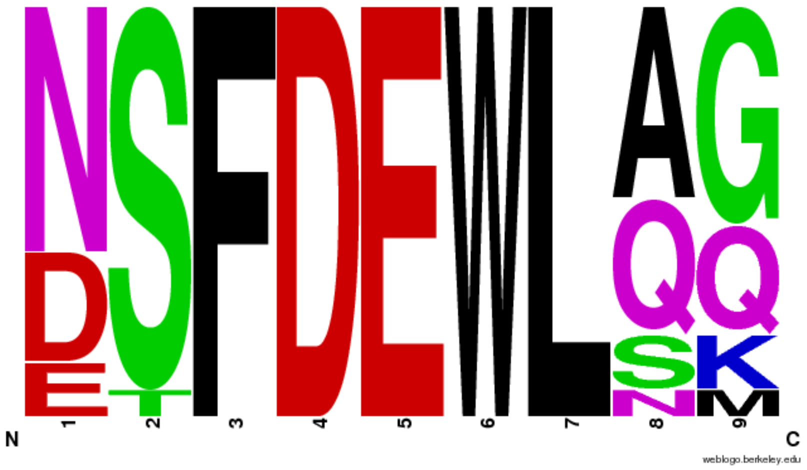

'LAEYH' extension library

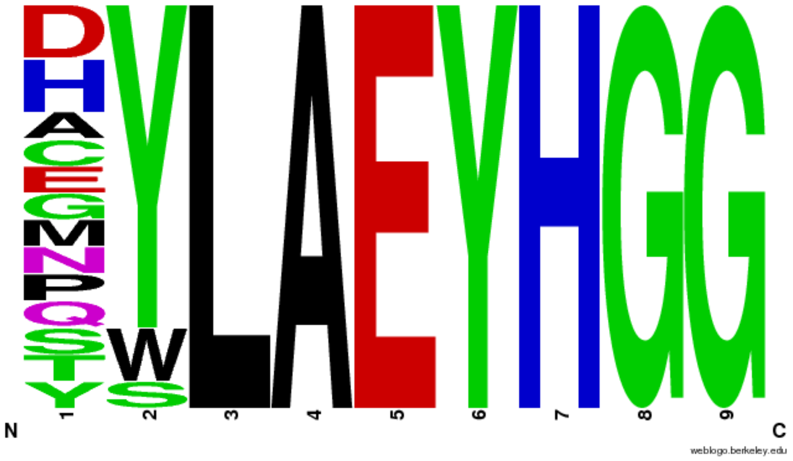

‘PAWAH’ extension library

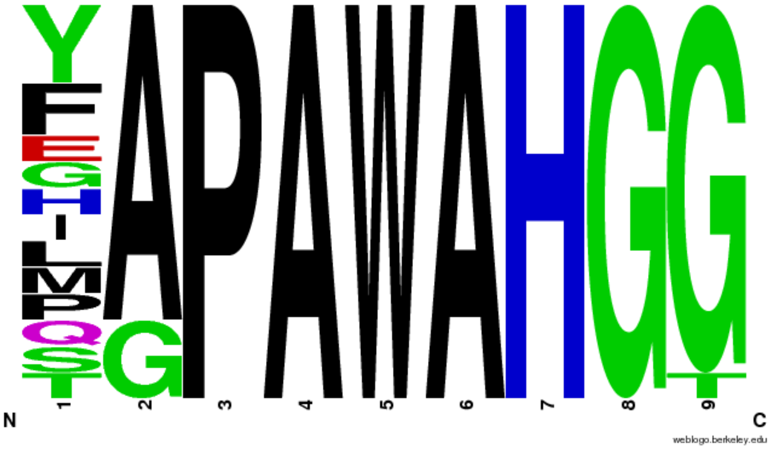

‘lanvd’ extension library

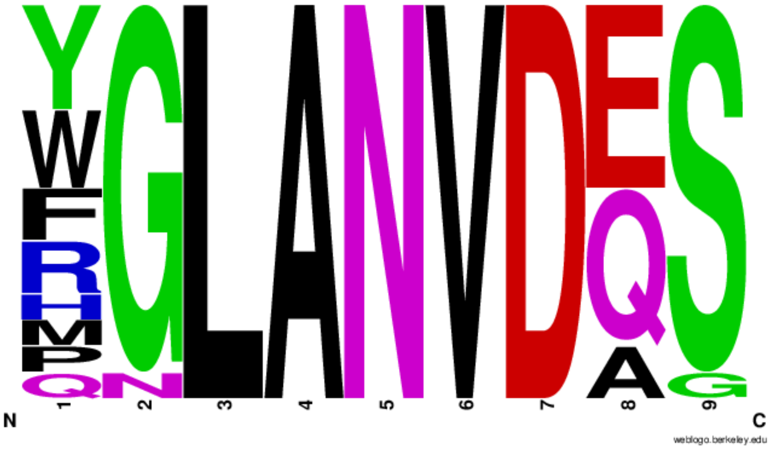

‘wqeea’ extension library

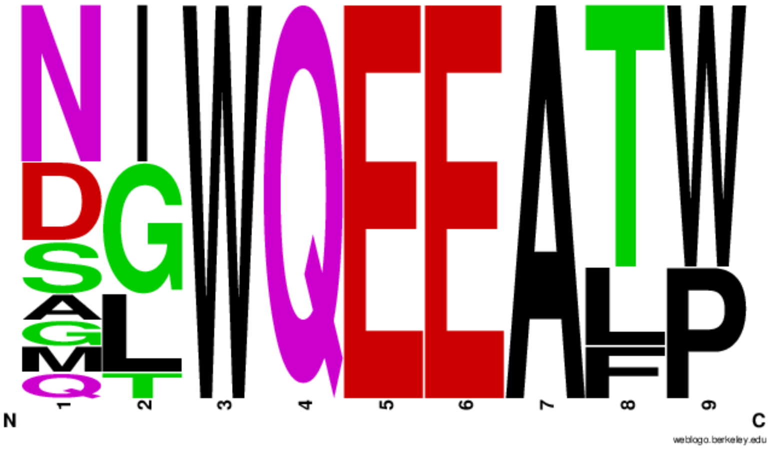

Supplementary Figure 3. Cyclic versus linear fluorescence signal intensity for the peptide library XXXXU bound to streptavidin-Cy5.

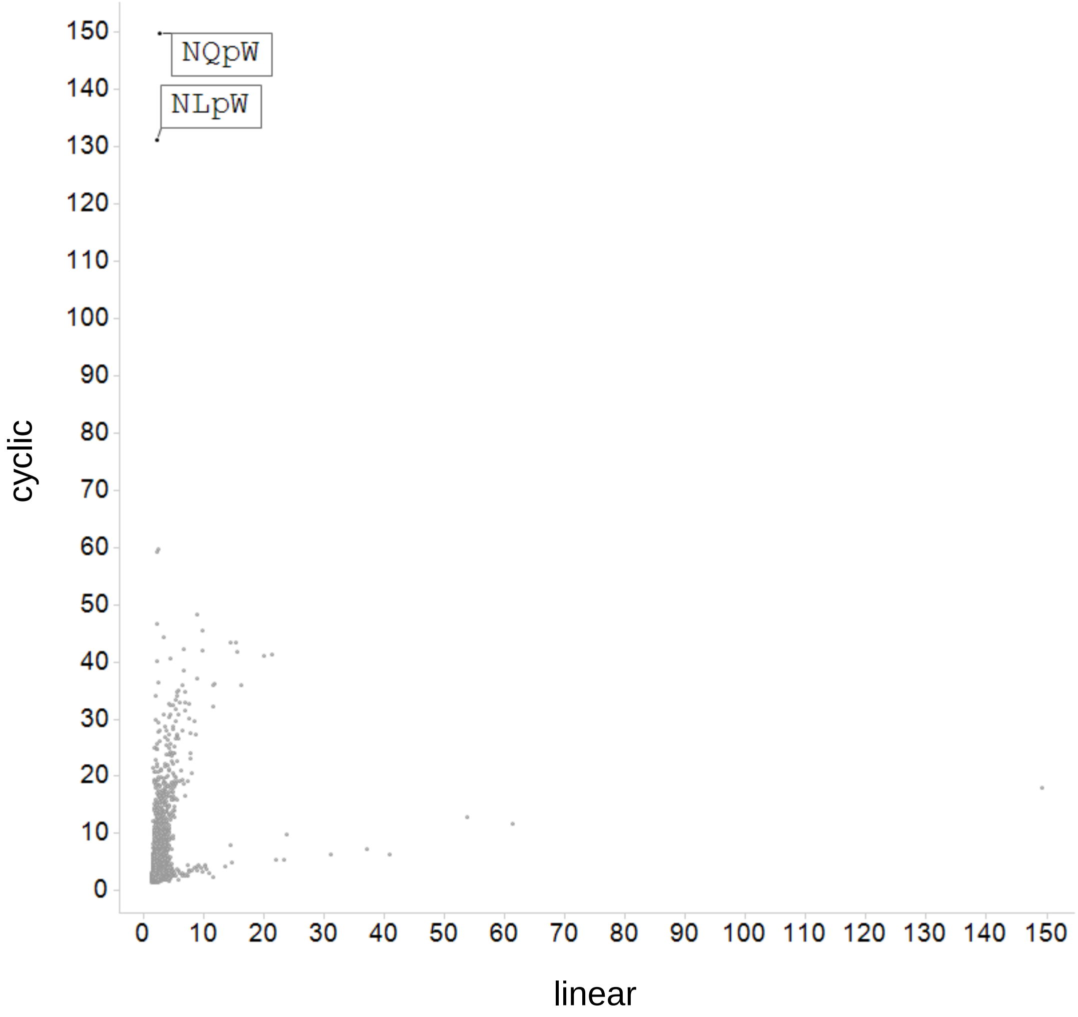

Supplementary Figure 4. SPR sensorgrams for interaction between surface immobilized streptavidin and L-, D- and cyclic L/D- peptides listed in Table 1. **A**. NH_2-SAWSHPQFEK-COOH L-peptide (Strep-tag II HPQ); **B**, NH_2-EWVHPQFEQKAK-amide L-peptide; **C**, NH_2-GNSFDDWLASKG-amide L-peptide; **D**, NH_2-AFPDYLAEYHGG-amide L-peptide; **E**, NH_2-RDPAPAWAHGGG-amide L-peptide; **F**, NH_2-GyGlanvdessG-amide D-peptide; **G**, NH_2-Gdlwqheatwkkq-amide D-peptide; **H**, NH_2-GGwhdeatwkpG-amide D-peptide; **I**, NQpWQ cyclic peptide. For peptides with 'fast on, fast off' kinetics (**A-B, I**), steady state affinity binding vs. concentration curves are shown and calculated equilibrium dissociation constant (K_d) indicated. GNSFDDWLASKG peptide (**C**) showed irreversible binding so binding parameters could not be determined. For other peptides (**D-H**), association rate constant (k_a), dissociation rate constant (k_d) and K_d were calculated using 1:1 binding model.

A. NH_2-SAWSHPQFEK-COOH (Strep-tag II HPQ)

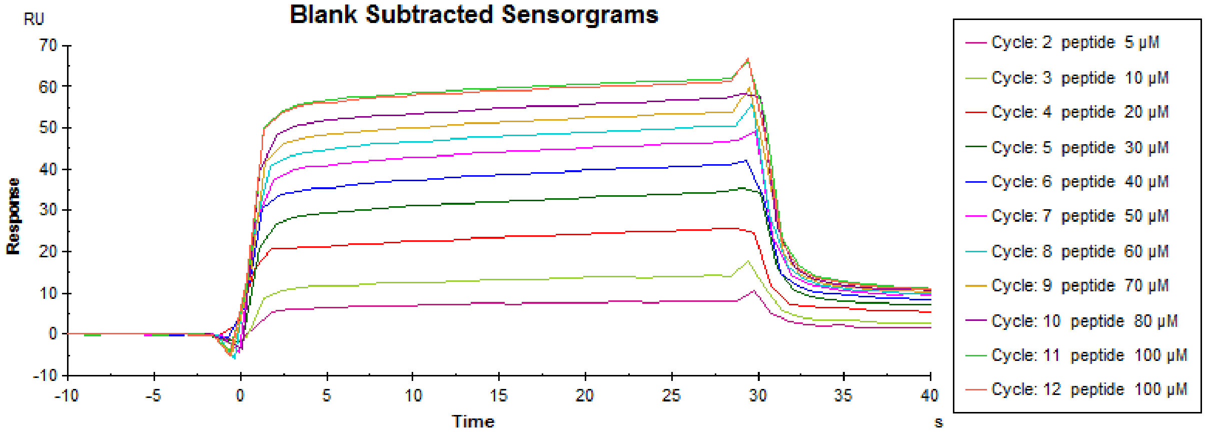

Steady state affinity curve, K_d 49.8 µM

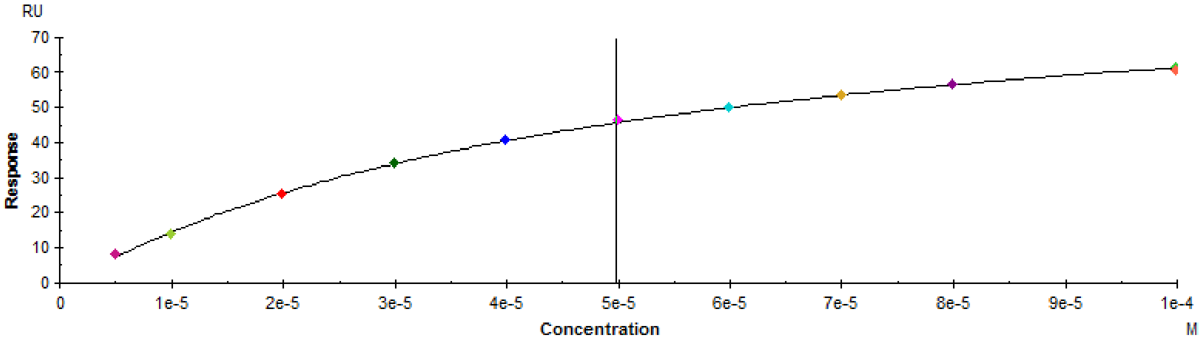

B, NH_2-EWVHPQFEQKAK-amide

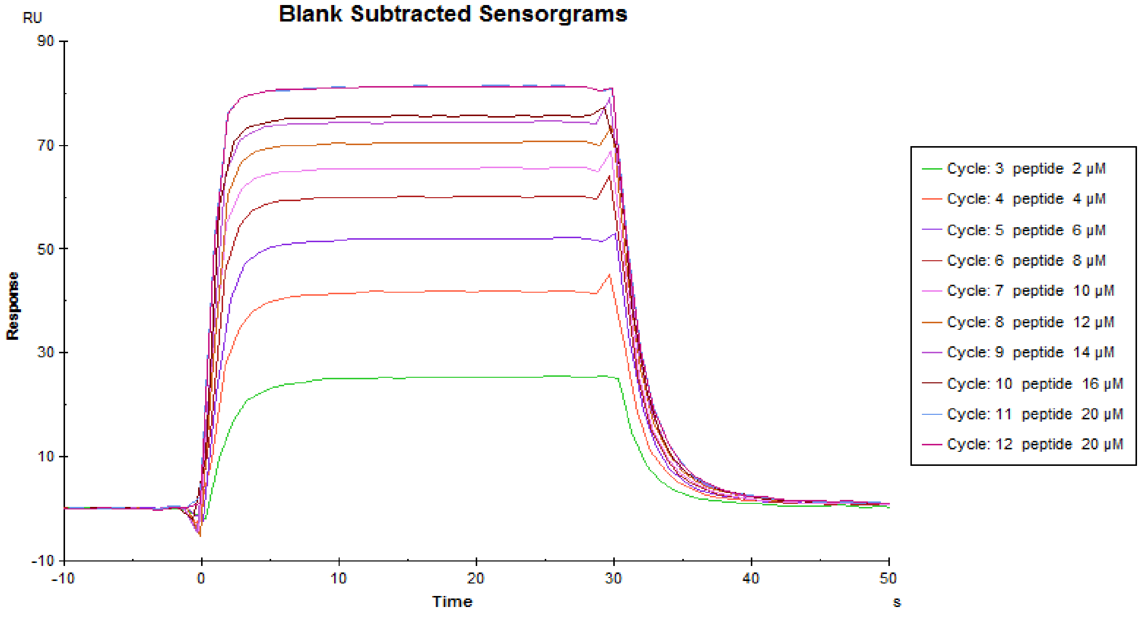

Steady state affinity curve, K_d 5.7 µM

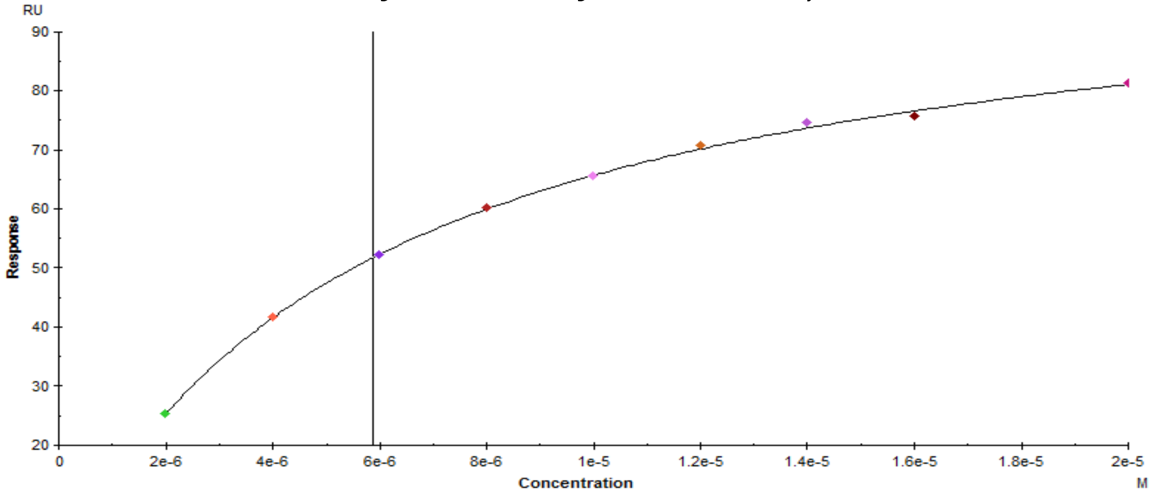

C, NH_2-GNSFDDWLASKG-amide

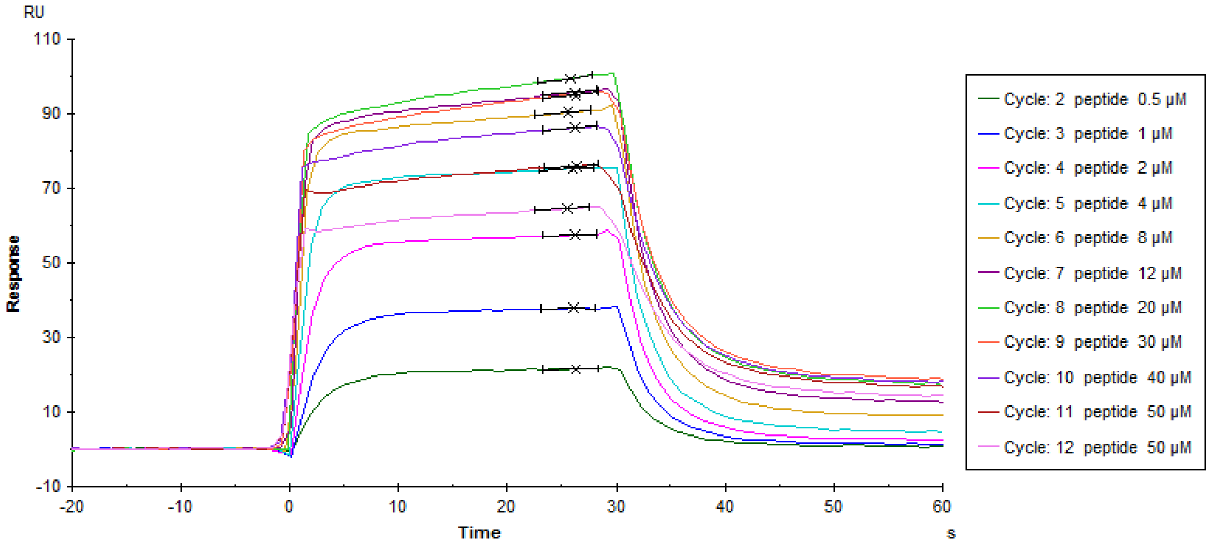

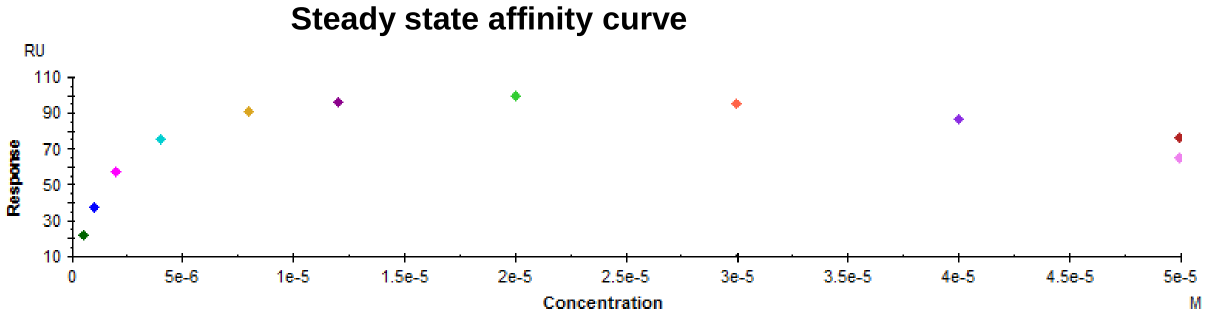

D, NH_2-AFPDYLAEYHGG-amide

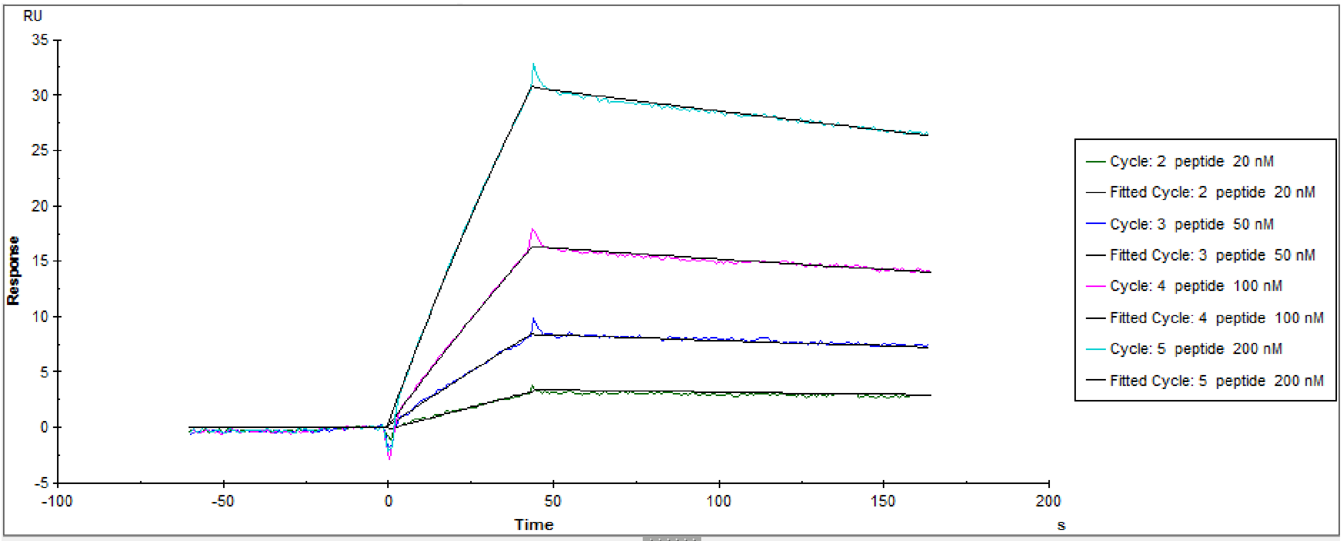

Quality Control | Report | Residuals | Parameters

Curve	ka (1/Ms)	kd (1/s)	KD (M)	Rmax (RU)	Conc (M)	tc	Flow (ul/min)	kt (RU/Ms)	RI (RU)	Chi² (RU²)	U-value
	3.007E+4	0.001278	4.248E-8	136.7		1.073E+8				0.101	2
**Cycle: 2 20 nM**					2.000E-8		30.00	3.335E+8	-0.1694		
**Cycle: 3 50 nM**					5.000E-8		30.00	3.335E+8	0.1336		
**Cycle: 4 100 nM**					1.000E-7		30.00	3.335E+8	0.1456		
**Cycle: 5 200 nM**					2.000E-7		30.00	3.335E+8	0.4177		

E, NH_2-RDPAPAWAHGGG-amide

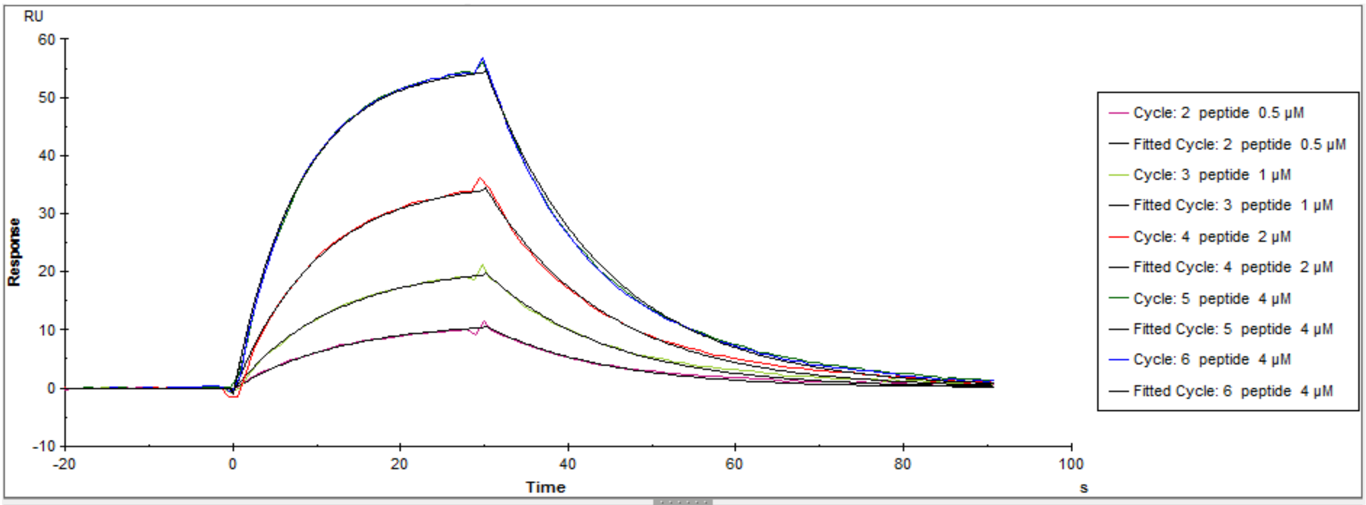

Quality Control | Report | Residuals | Parameters

Curve	ka (1/Ms)	kd (1/s)	KD (M)	Rmax (RU)	Conc (M)	tc	Flow (ul/min)	kt (RU/Ms)	RI (RU)	Chi² (RU²)	U-value
	1.492E+4	0.06956	4.661E-6	122.3		3.024E+10				0.228	1
Cycle: 2 0.5 µM					5.000E-7		30.00	9.395E+10	-0.2638		
Cycle: 3 1 µM					1.000E-6		30.00	9.395E+10	-0.4141		
Cycle: 4 2 µM					2.000E-6		30.00	9.395E+10	-0.8456		
Cycle: 5 4 µM					4.000E-6		30.00	9.395E+10	-1.045		
Cycle: 6 4 µM					4.000E-6		30.00	9.395E+10	-1.025		

F, NH_2-GyGlanvdessG-amide D-peptide

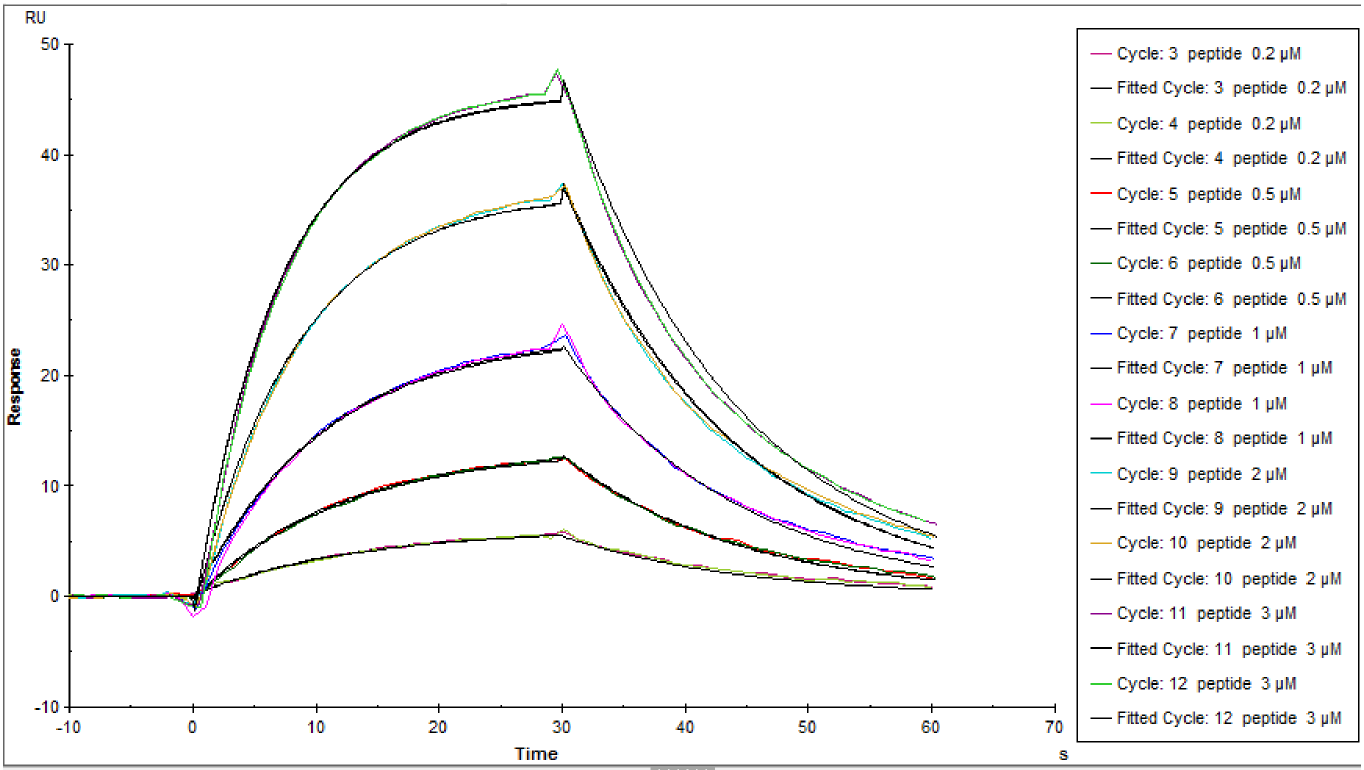

Quality Control | Report | Residuals | Parameters

Curve	ka (1/Ms)	kd (1/s)	KD (M)	Rmax (RU)	Conc (M)	tc	Flow (ul/min)	kt (RU/Ms)	RI (RU)	Chi² (RU²)	U-value
	2.482E+4	0.07125	2.870E-6	93.39		1.643E+9				0.206	1
**Cycle: 3 0.2 µM**					2.000E-7		30.00	5.104E+9	0.1173		
**Cycle: 4 0.2 µM**					2.000E-7		30.00	5.104E+9	0.06025		
**Cycle: 5 0.5 µM**					5.000E-7		30.00	5.104E+9	-0.2611		
**Cycle: 6 0.5 µM**					5.000E-7		30.00	5.104E+9	-0.4367		
**Cycle: 7 1 µM**					1.000E-6		30.00	5.104E+9	-0.3845		
**Cycle: 8 1 µM**					1.000E-6		30.00	5.104E+9	-0.5493		
**Cycle: 9 2 µM**					2.000E-6		30.00	5.104E+9	-1.797		
**Cycle: 10 2 µM**					2.000E-6		30.00	5.104E+9	-1.821		
**Cycle: 11 3 µM**					3.000E-6		30.00	5.104E+9	-2.241		
**Cycle: 12 3 µM**					3.000E-6		30.00	5.104E+9	-2.362		

G, NH_2-Gdlwqheatwkkq-amide D-peptide

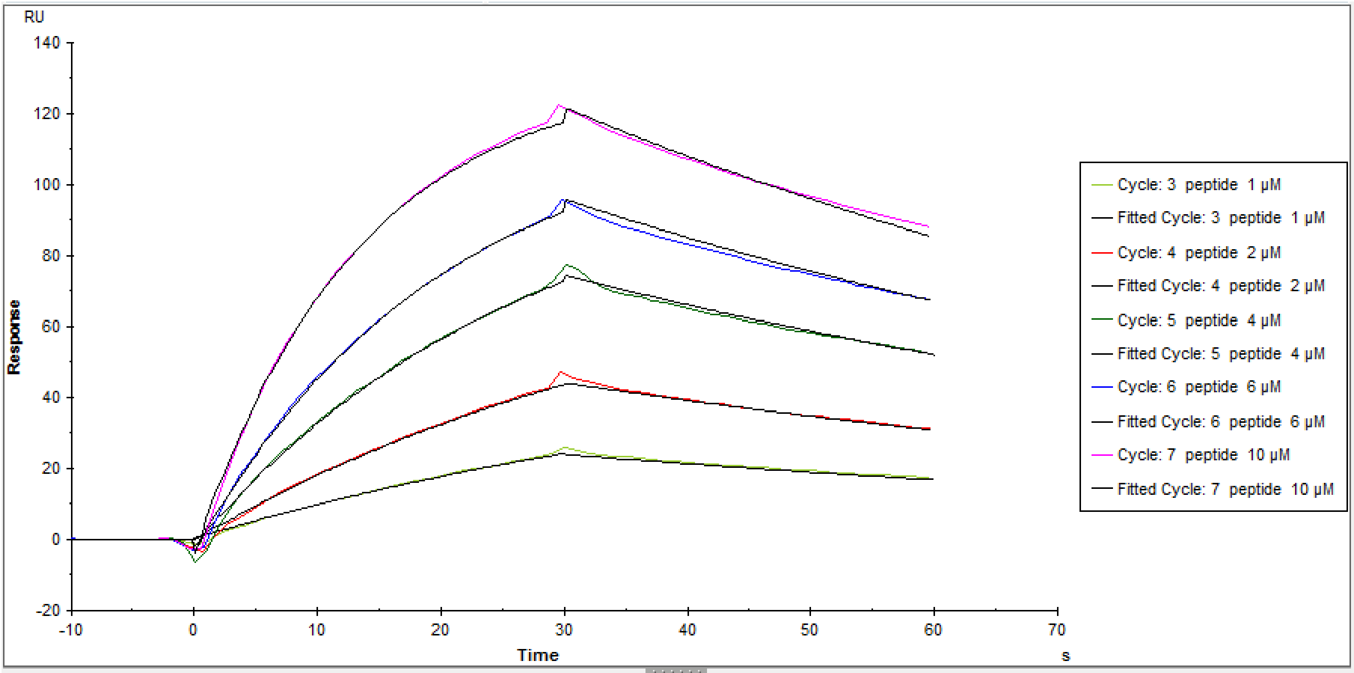

Quality Control | Report | Residuals | Parameters

Curve	ka (1/Ms)	kd (1/s)	KD (M)	Rmax (RU)	Conc (M)	tc	Flow (ul/min)	kt (RU/Ms)	RI (RU)	Chi² (RU²)	U-value
	6485	0.01191	1.837E-6	159.9		4.501E+18				0.710	1
Cycle: 3 1 µM					1.000E-6		30.00	1.398E+19	0.2926		
Cycle: 4 2 µM					2.000E-6		30.00	1.398E+19	-0.2846		
Cycle: 5 4 µM					4.000E-6		30.00	1.398E+19	-1.747		
Cycle: 6 6 µM					6.000E-6		30.00	1.398E+19	-3.465		
Cycle: 7 10 µM					1.000E-5		30.00	1.398E+19	-4.078		

H, NH_2-GGwhdeatwkpG-amide

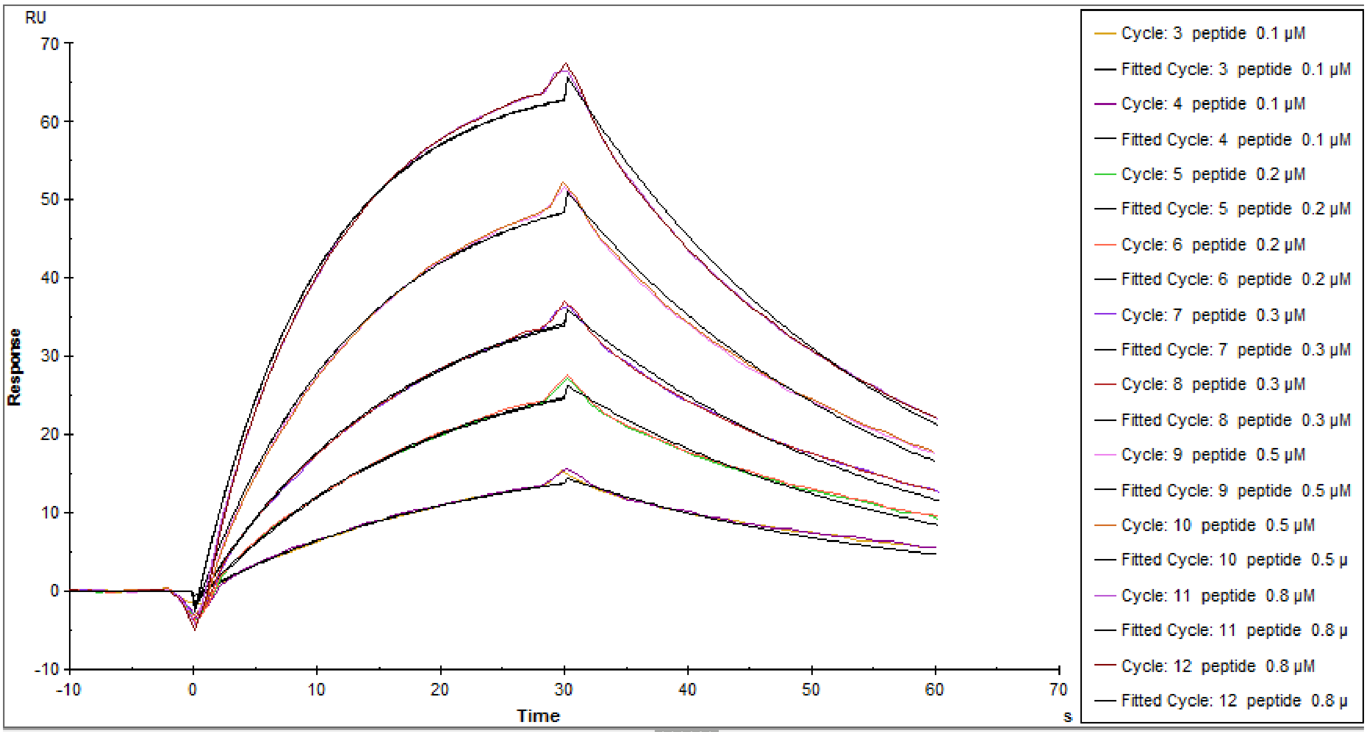

Quality Control | Report | Residuals | Parameters

Curve	ka (1/Ms)	kd (1/s)	KD (M)	Rmax (RU)	Conc (M)	tc	Flow (ul/min)	kt (RU/Ms)	RI (RU)	Chi² (RU²)	U-value
	8.071E+4	0.03797	4.704E-7	110.5		4.110E+10				0.441	1
**Cycle: 3 0.1 µM**					1.000E-7		30.00	1.277E+11	-0.7818		
**Cycle: 4 0.1 µM**					1.000E-7		30.00	1.277E+11	-0.7464		
**Cycle: 5 0.2 µM**					2.000E-7		30.00	1.277E+11	-1.906		
**Cycle: 6 0.2 µM**					2.000E-7		30.00	1.277E+11	-1.700		
**Cycle: 7 0.3 µM**					3.000E-7		30.00	1.277E+11	-2.452		
**Cycle: 8 0.3 µM**					3.000E-7		30.00	1.277E+11	-2.216		
**Cycle: 9 0.5 µM**					5.000E-7		30.00	1.277E+11	-3.089		
**Cycle: 10 0.5 µM**					5.000E-7		30.00	1.277E+11	-3.130		
**Cycle: 11 0.8 µM**					8.000E-7		30.00	1.277E+11	-3.624		
**Cycle: 12 0.8 µM**					8.000E-7		30.00	1.277E+11	-3.519		

I, NQpWQ cyclic peptide

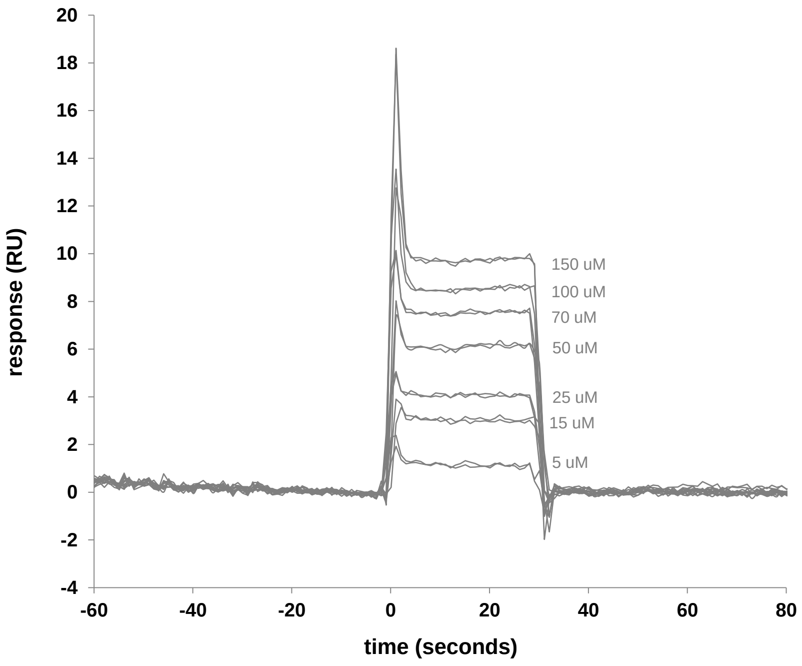

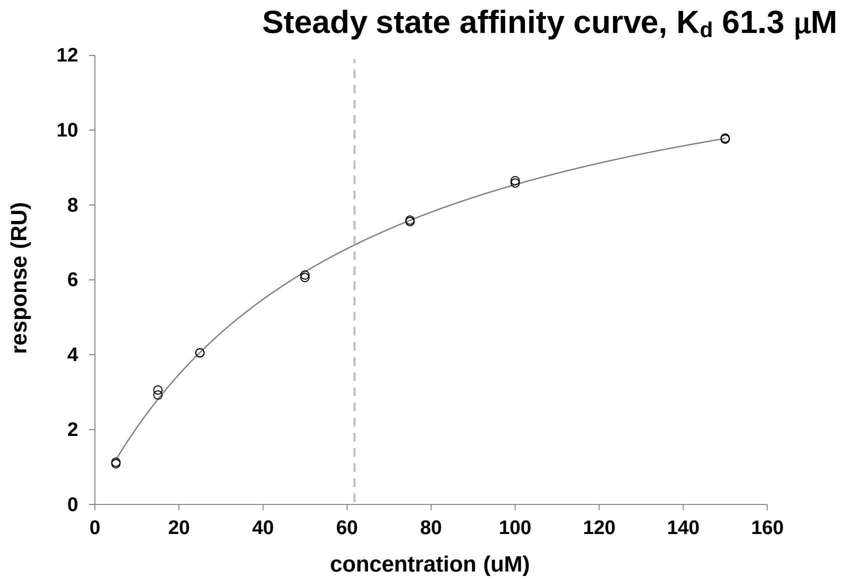

Supplementary Figure 5. Specificity plot and co-crystal structure with streptavidin for AFPDYLAEYHGG L-peptide. (A) Double substitution plot for AFPDYLAQYHGG peptide with Glu amino acid fixed at position Q8 showing signal intensity for 20 amino acid substitutions for each peptide position. The color coding scheme for the bars representing signal intensity is the same as in Figure 2. (B) Co-crystal structure with streptavidin for AFPDYLAEYHGG peptide. The dashed lined show interactions between peptide (green) and streptavidin (grey) key amino acids at 4A distance. Biotin-binding pocket of streptavidin shown as surface.

The AFPDYLAEYHGG peptide exhibits the highest affinity for streptavidin in SPR measurements (Table 1) and shows tight interaction network with streptavidin. The backbone nitrogen and carbonyl of Ala1 form hydrogen bonds with Ser112 and Ser88, respectively. Phe2 is involved in hydrophobic interactions with Leu110 and Leu124. Structural- and substitution-critical amino acids (Fig. 2) include: (1) Tyr5, which stacks against the sidechain of Arg84; (2) Tyr9, which forms a hydrogen bond with Ser52 in loop3/4; (3) His10, which forms a hydrogen bond with Thr90; and (4) Gly11, which interacts with Leu25 and Asp128.

A

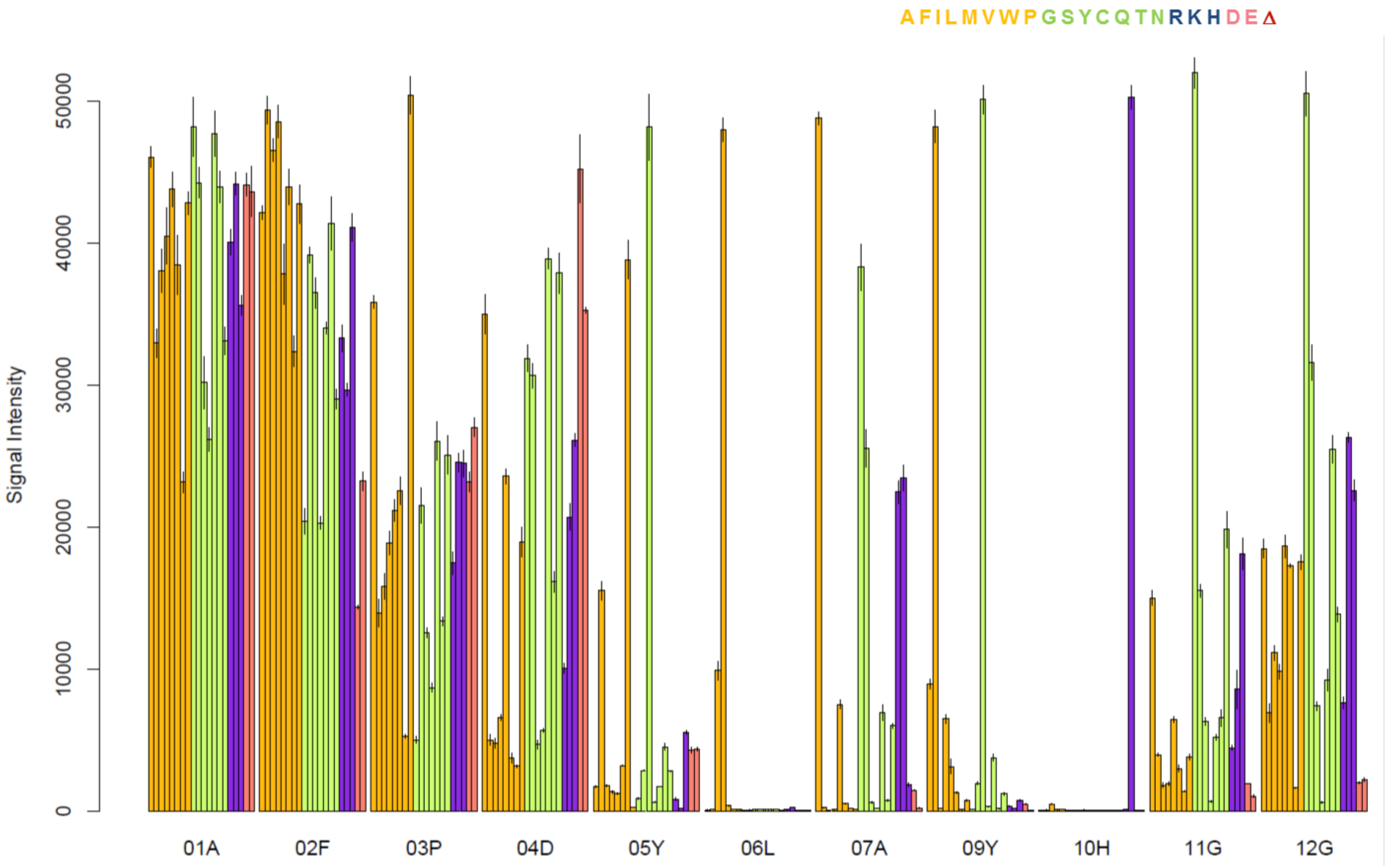

B

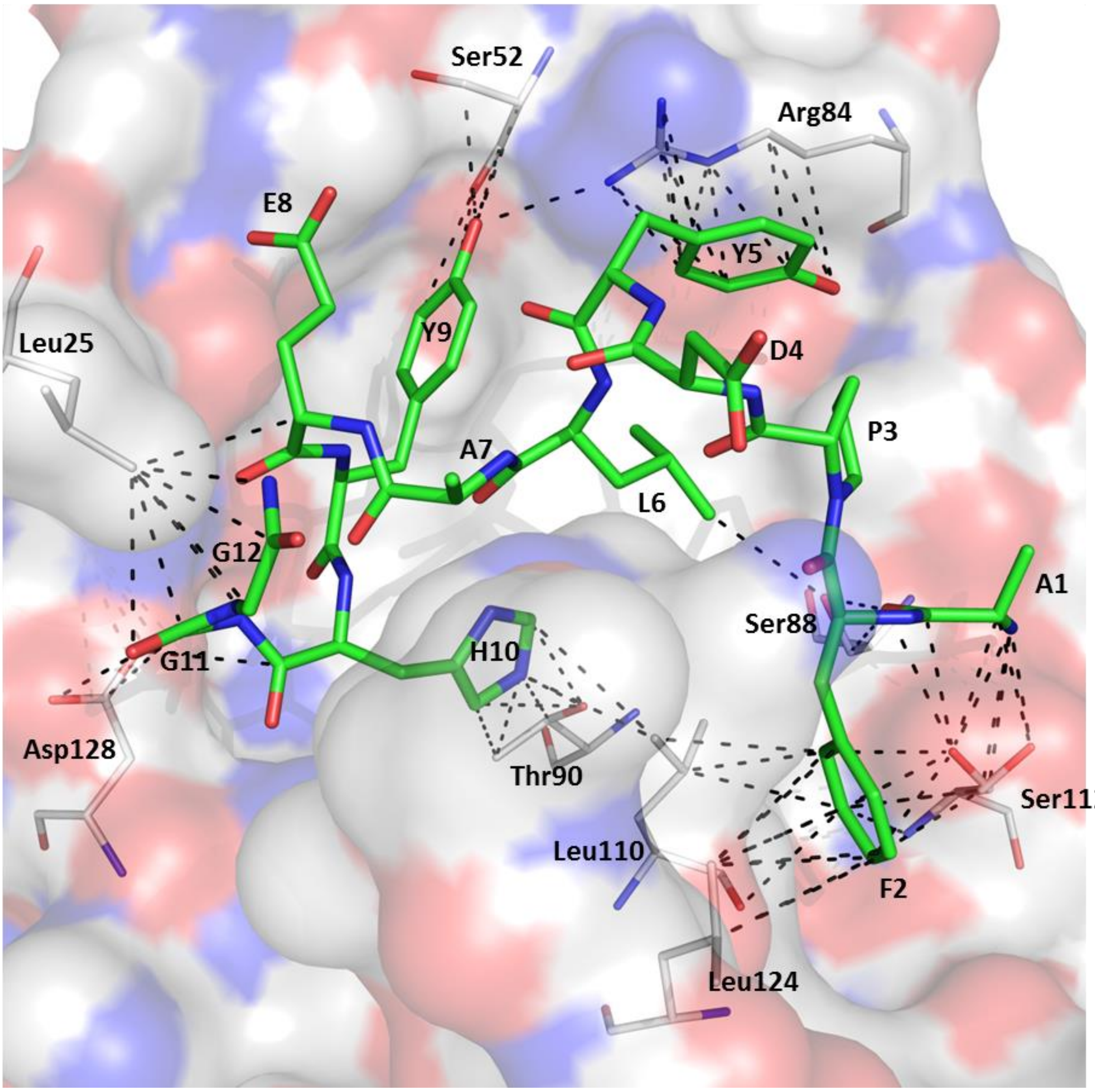

Ser52
Arg84
E8
Y5
Y9
Leu25
D4
P3
A7
L6
G12
A1
Ser88
H10
G11
Asp128
Thr90
Ser112
Leu110
F2
Leu124

Supplementary Figure 6. Specificity plot and co-crystal structure with streptavidin for the RDPAPAWAHGGG L-peptide. (A) Substitution plot showing signal intensity for 20 amino acid substitutions and a deletion for each peptide position. The color coding scheme for the bars representing signal intensity is the same as in Figure 2. (B) The dashed lines show interactions between the peptide (green) and streptavidin (orange) key amino acids (sticks) at 4 Å distance. The biotin binding pocket of streptavidin is shown as a surface. The peptide amino acids are shown in the single-letter amino acid code and streptavidin ones in the three-letter amino acid code.

The backbone carbonyl oxygen of Pro3 of the RDPAPAWAHGGG peptide forms a hydrogen bond with Ser88, and Pro5 overlaps with the position of Pro5 of the EWVHPQFEQKAK peptide and allows hydrogen bonds to form between the highly specific His9 of the peptide and Thr90 of streptavidin. Another invariant residue, Trp7, contributes to the binding by forming a van der Waals interaction with the backbone of Glu44 and Ser45 of loop 3/4. The short hydrophobic sidechain of the highly specific Ala8 is structurally critical for an edge-to-face interaction with Trp79. The C-terminal region of the RDPAPAWAHGGG peptide extends to a neighboring streptavidin subunit for additional van der Waals contacts with Trp120.

A

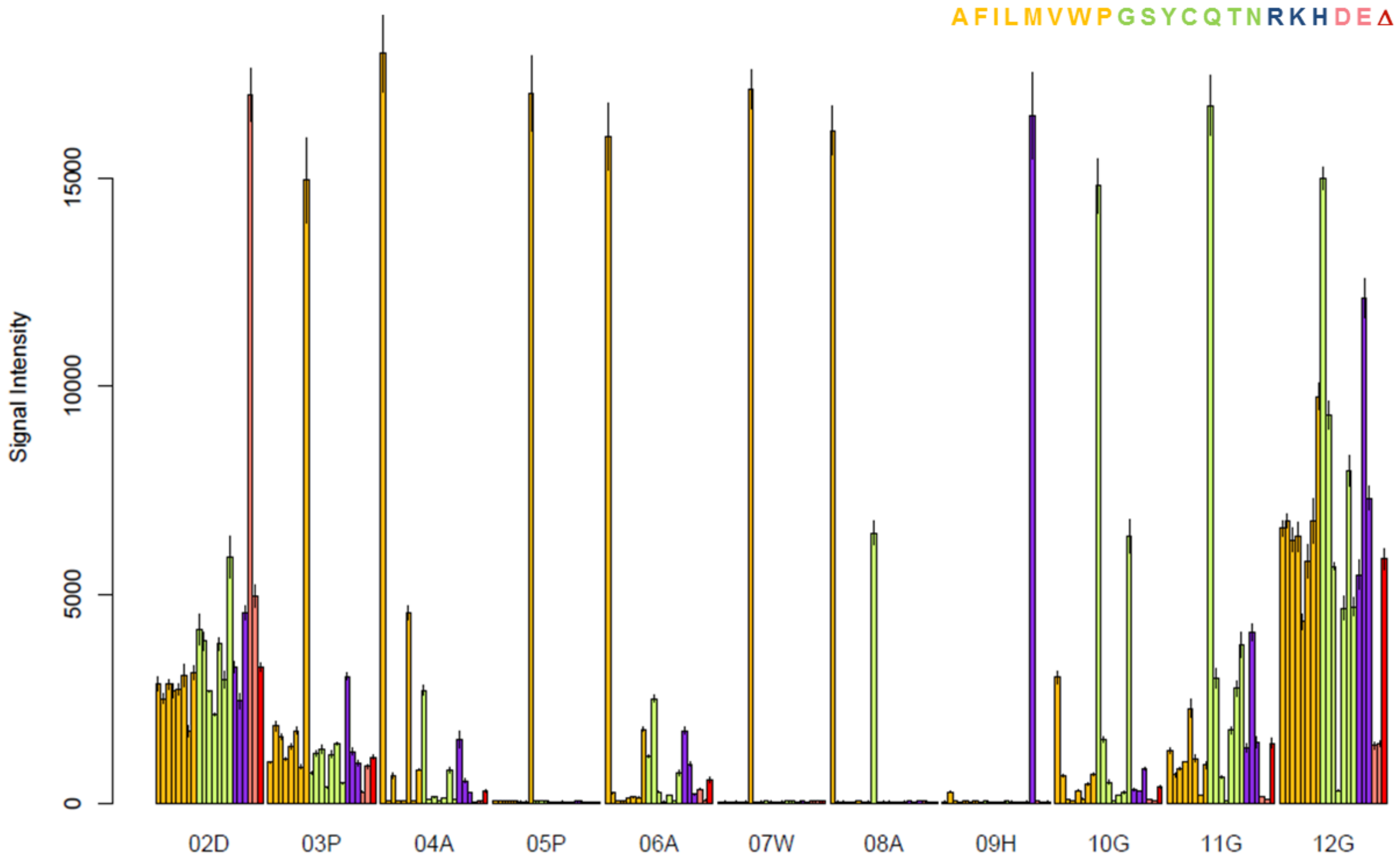

B

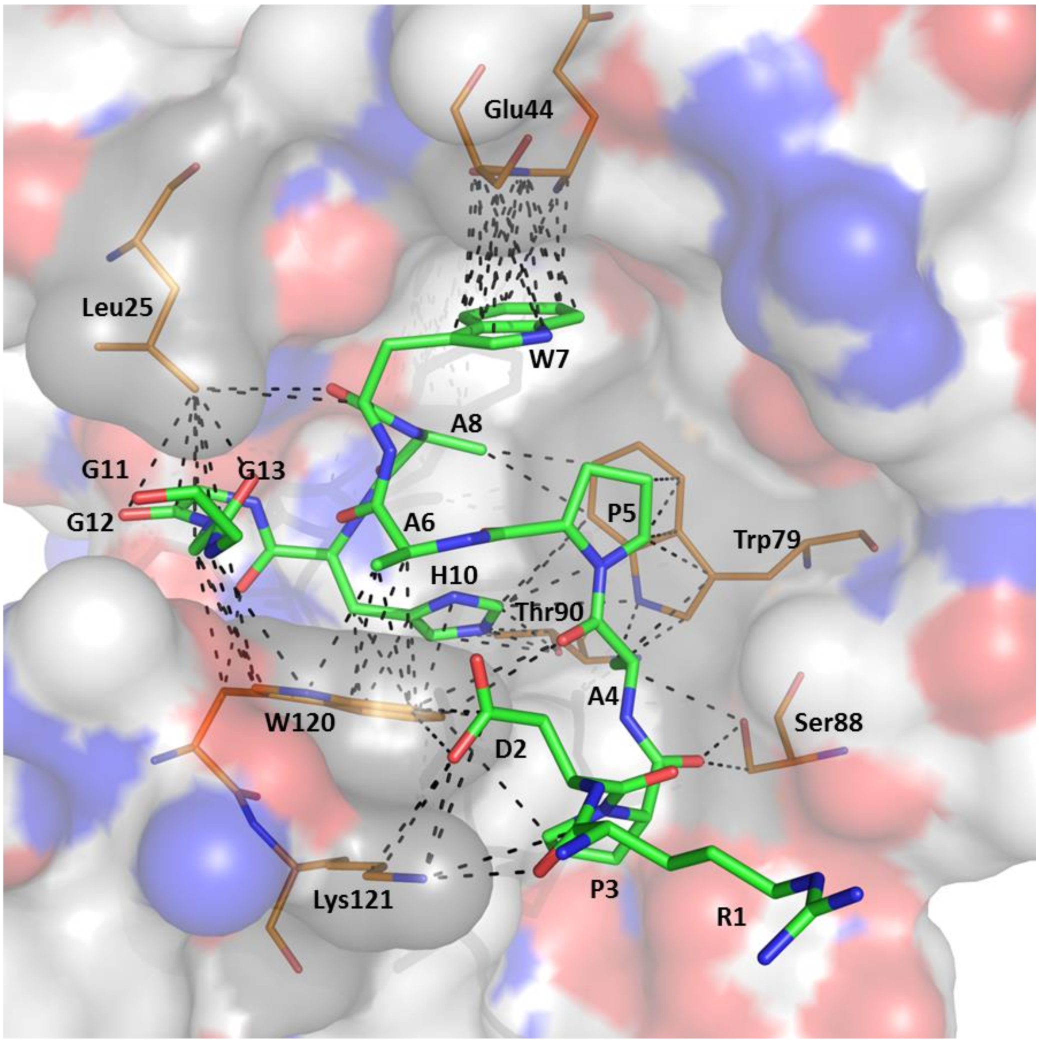
Glu44
Leu25
W7
A8
G11
G13
G12
A6
P5
Trp79
H10
Thr90
A4
Ser88
W120
D2
Lys121
P3
R1

Supplementary Figure 7. Co-crystal structure and specificity plots with streptavidin for Gdlwqheatwkkq and GGwhdeatwkpG D-peptides. (A) The dashed lines show interactions between the GGwhdeatwkpG peptide (green) and streptavidin (grey) key amino acids (sticks) at 4 Å distance. The superimposed structure of the Gdlwqheatwkkq peptide is shown in yellow. Peptide amino acids are indicated by the single-letter amino acid code and streptavidin amino acids are indicated by the three-letter amino acid code. (B) Substitution plot for the “wheea” region of the d5e variant of the GGwhdeatwkpG peptide. (C) Substitution plot for the “wqhea” region of the Gdlwqheatwkkq peptide. The color coding scheme for the bars representing signal intensity is the same as in Figure 2.

A

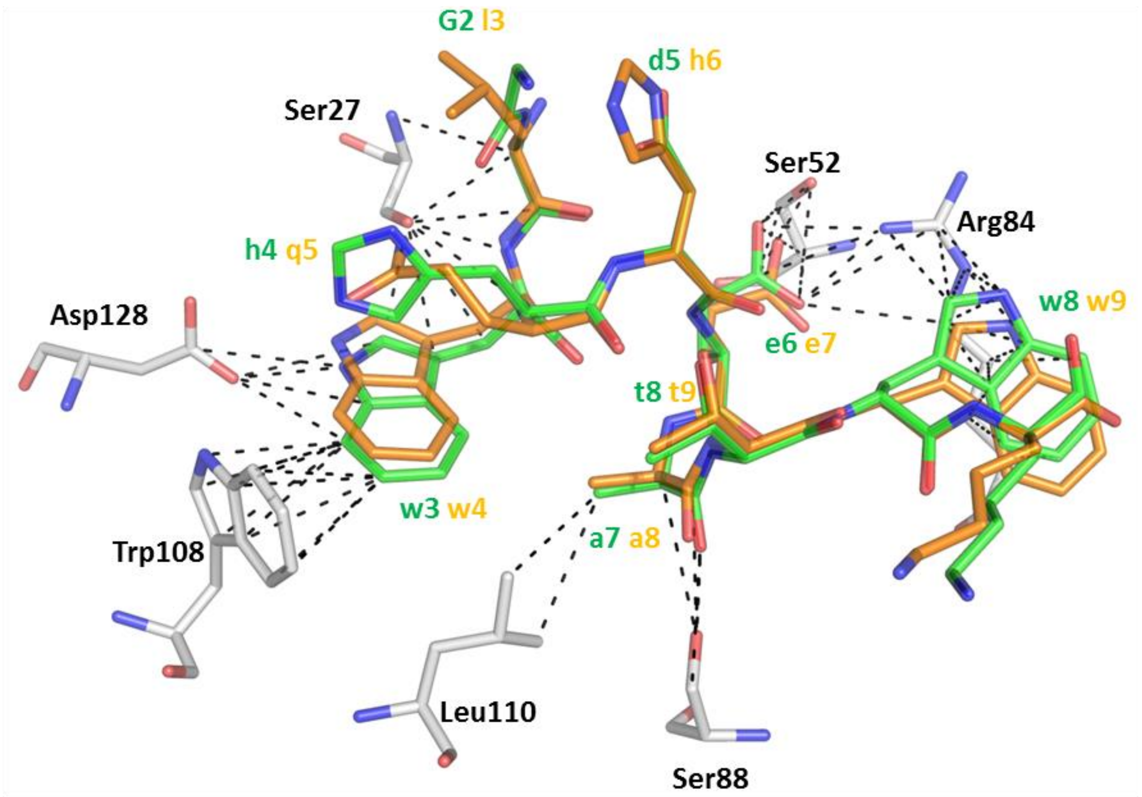

B

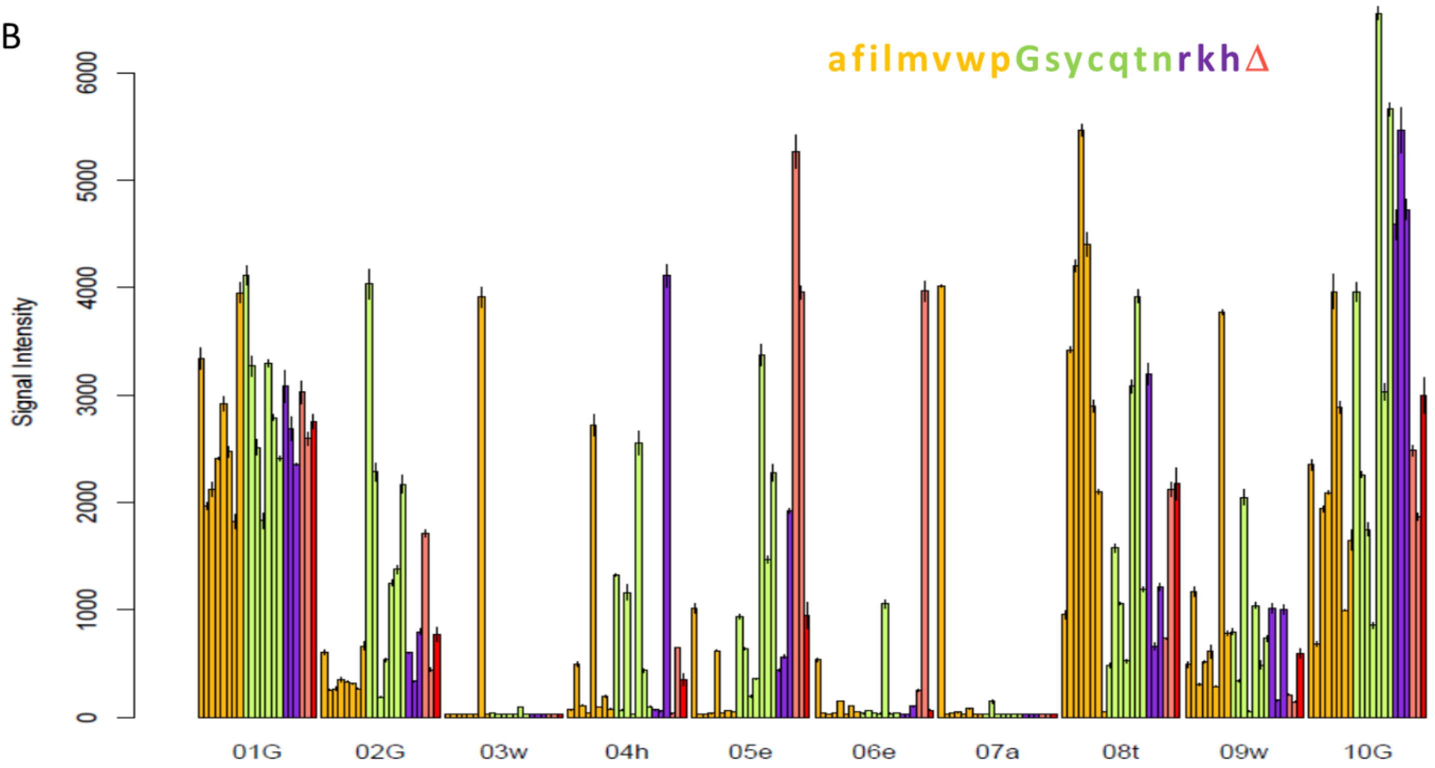
afilmvwpGsycqtnrkhΔ
Signal Intensity
0
1000
2000
3000
4000
5000
6000
01G
02G
03w
04h
05e
06e
07a
08t
09w
10G

C

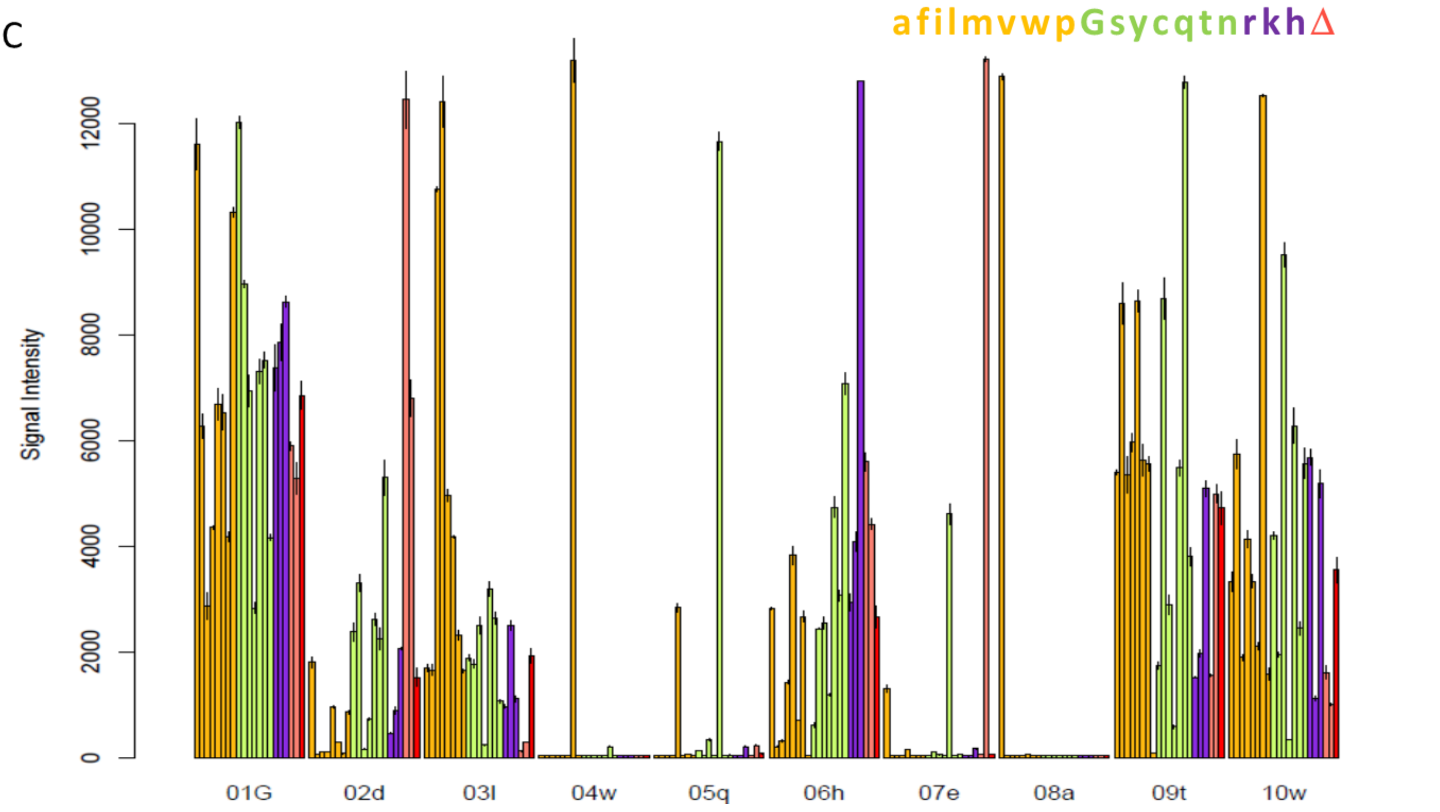
afilmvwpGsycqtnrkhΔ
Signal Intensity
0
2000
4000
6000
8000
10000
12000
01G
02d
03l
04w
05q
06h
07e
08a
09t
10w

Supplementary Figure 8. Specificity plot and co-crystal structure with streptavidin for the GyGlanvdessG D-peptide. (A) Substitution plot showing signal intensity for 20 amino acid substitutions and a deletion for each peptide position. The color coding scheme for the bars representing signal intensity is the same as in Figure 2. (B) The dashed lines show interactions between the peptide (green) and streptavidin (orange) key amino acids (sticks) at 4 Å distance. The biotin binding pocket of streptavidin is shown as a surface. Peptide amino acids are indicated by the single-letter amino acid code and streptavidin amino acids are indicated by the single-letter amino acid code.

The short, aliphatic sidechain of D-Ala5 is needed to fit into a small pocket near Trp79. D-Asn6, which closely overlaps with the Gln of the HPQ motif in the EWVHPQFEQKAK structure, is also indispensable because it is involved in a network of interactions with Thr90, Trp108, Leu110, and Trp120 from a neighboring subunit. Further, the D-Asp8 sidechain has the optimal length to form charge-charge interactions with Arg84.

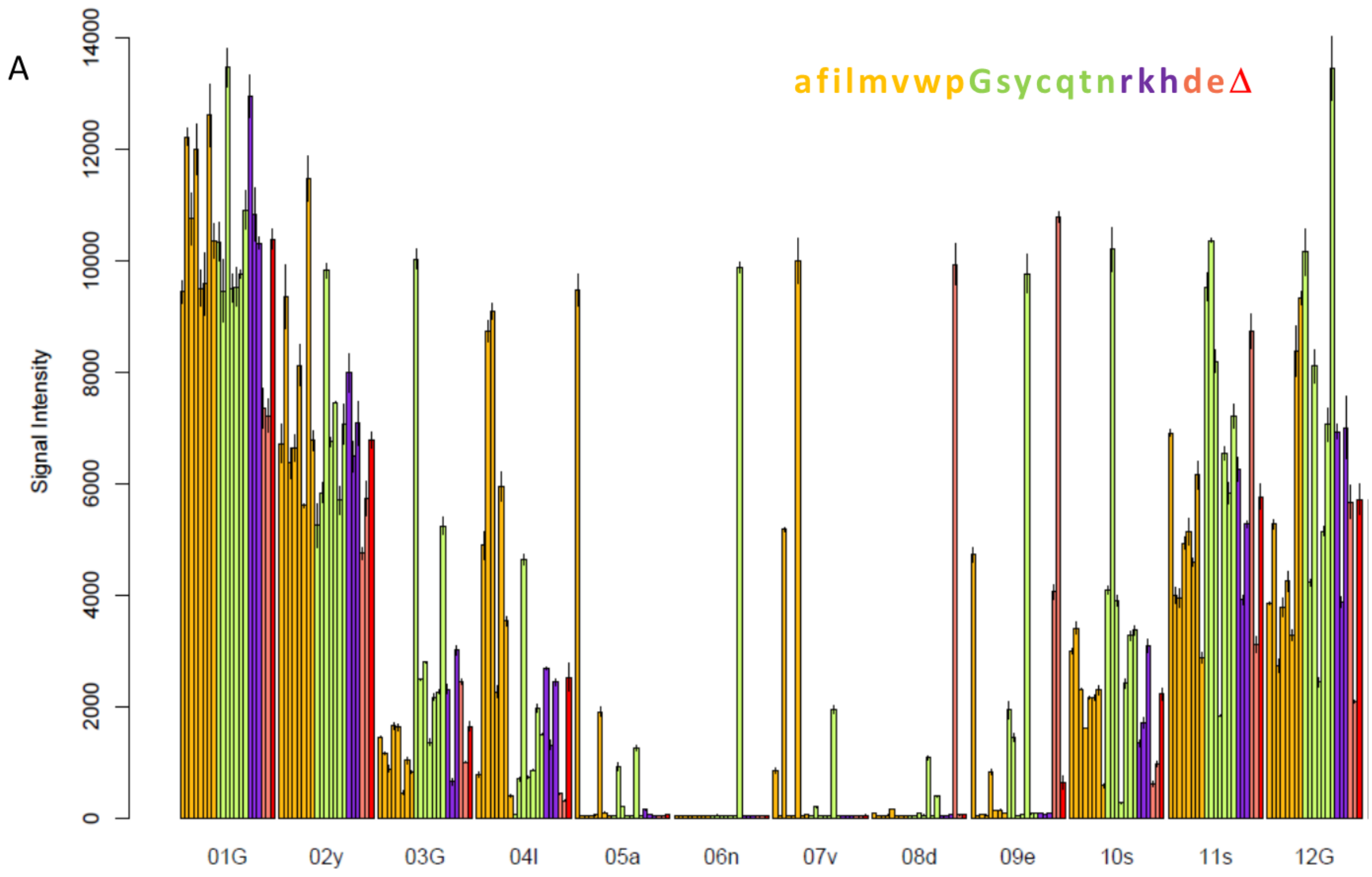

B

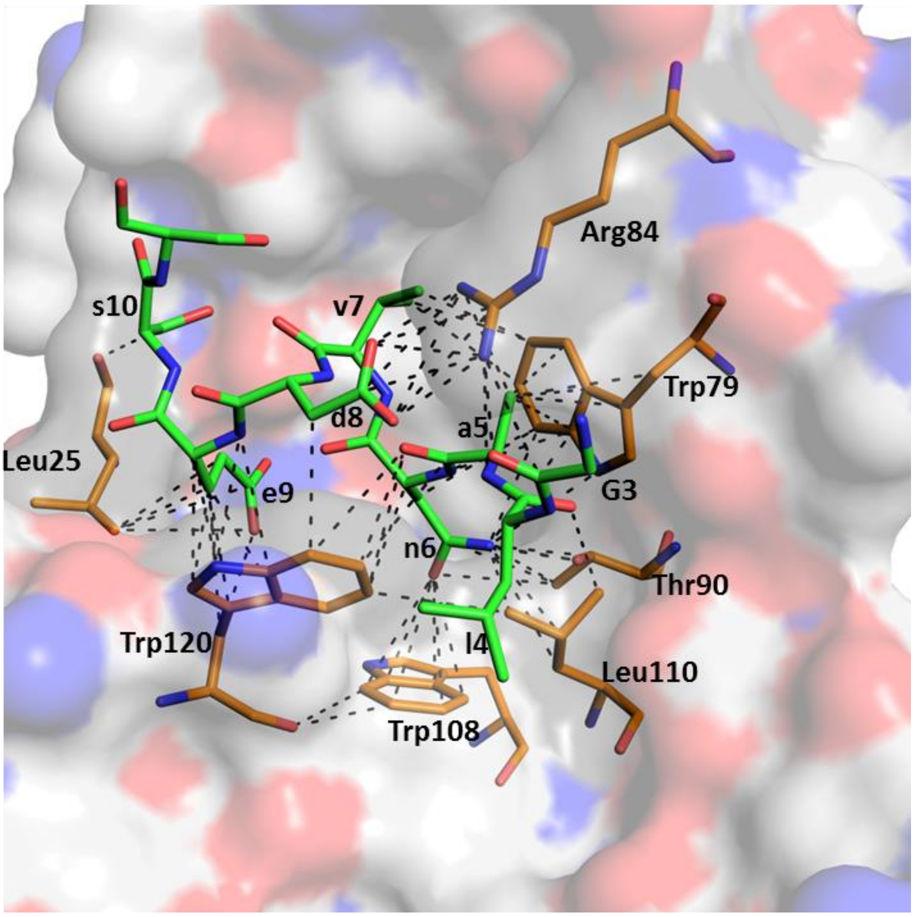

Arg84
s10
v7
Trp79
d8
a5
Leu25
e9
G3
n6
Thr90
Trp120
I4
Leu110
Trp108
